# Distributed Bayesian Computation and Self-Organized Learning in Sheets of Spiking Neurons with Local Lateral Inhibition

**DOI:** 10.1371/journal.pone.0134356

**Published:** 2015-08-18

**Authors:** Johannes Bill, Lars Buesing, Stefan Habenschuss, Bernhard Nessler, Wolfgang Maass, Robert Legenstein

**Affiliations:** 1 Institute for Theoretical Computer Science, TU Graz, Graz, Austria; 2 Department of Statistics, Columbia University, New York, New York, United States of America; 3 Frankfurt Institute for Advanced Studies, Frankfurt am Main, Germany; Georgia State University, UNITED STATES

## Abstract

During the last decade, Bayesian probability theory has emerged as a framework in cognitive science and neuroscience for describing perception, reasoning and learning of mammals. However, our understanding of how probabilistic computations could be organized in the brain, and how the observed connectivity structure of cortical microcircuits supports these calculations, is rudimentary at best. In this study, we investigate statistical inference and self-organized learning in a spatially extended spiking network model, that accommodates both local competitive and large-scale associative aspects of neural information processing, under a unified Bayesian account. Specifically, we show how the spiking dynamics of a recurrent network with lateral excitation and local inhibition in response to distributed spiking input, can be understood as sampling from a variational posterior distribution of a well-defined implicit probabilistic model. This interpretation further permits a rigorous analytical treatment of experience-dependent plasticity on the network level. Using machine learning theory, we derive update rules for neuron and synapse parameters which equate with Hebbian synaptic and homeostatic intrinsic plasticity rules in a neural implementation. In computer simulations, we demonstrate that the interplay of these plasticity rules leads to the emergence of probabilistic local experts that form distributed assemblies of similarly tuned cells communicating through lateral excitatory connections. The resulting sparse distributed spike code of a well-adapted network carries compressed information on salient input features combined with prior experience on correlations among them. Our theory predicts that the emergence of such efficient representations benefits from network architectures in which the range of local inhibition matches the spatial extent of pyramidal cells that share common afferent input.

## Introduction

Humans and animals perceive their environment through a stream of data from various high-dimensional sensory modalities. Successful behavior requires that the individual dimensions of this data stream are aligned with one another and integrated into a compact representation that promotes rapid decision making and generalization. Typically, the available sensory information on which decisions have to be based is noisy, unreliable and incomplete. Hence, it is essential that such representations respect the statistical nature of sensory data and that knowledge about statistical and causal relations among events in the external world are taken into account when a representation is generated. In recent years, Bayesian inference has been identified in cognitive science as a powerful normative framework for the description of cognitive processes in face of uncertainty in humans [1–3] and animals [4]. The Bayesian framework has also been successfully employed for a formal description of learning, for instance in perceptual [5, 6] and sensorimotor [7, 8] learning tasks.

In the Bayesian framework, quantities of interest are formally treated as random variables (RVs), and beliefs about their current values are formalized as probability distributions over these RVs [9]. Typically, one distinguishes between observations *y*_*i*_, *i* = 1, .., *N*, representing directly observable variables, and latent variables *z*_*k*_, *k* = 1, .., *K* which cannot be observed directly. Latent variables represent abstract features and concepts that allow to structure and conceive the given input. As an everyday example, the high dimensional vector ***y*** = (*y*_1_, .., *y*_*N*_)^⊺^ of input RVs could summarize the entire sensory stream from the visual, auditory, and vestibular system while driving by bike in a city during rush hour. Latent variables ***z*** = (*z*_1_, .., *z*_K_)^⊺^ could represent streets, cars, and pedestrians, or even more abstract features, such as estimated velocities, potential threats or anticipated actions. Thus, the latent variables ***z*** form a compact representation of relevant aspects of the scene which is typically more stable and informative than individual local observables *y*_*i*_. In this manner, local observations *y*_*i*_, which are often unreliable, e.g., due to noise or an occluding obstacle, can be integrated into a consistent global interpretation.

In a Bayesian framework, all knowledge about statistical dependencies between RVs is represented in terms of a probability distribution *p* (***z***, ***y*** ∣ ***θ***), where the parameter vector ***θ*** shapes the statistical model of the environment. The aim of Bayesian inference is to infer a belief over the possible states of the latent variables ***z*** for the given observations ***y***. More precisely, Bayesian inference is the calculation of the posterior distribution *p* (***z*** ∣ ***y***, ***θ***) over the latent variables ***z*** given the input ***y***. In ambiguous situations (e.g. when estimating the speed of a car), the posterior *p* (***z*** ∣ ***y***, ***θ***) provides not only the most likely interpretation of the input, but rather the probabilities of the most likely and all alternative interpretations. Finally, *p* (***z*** ∣ ***y***, ***θ***) can also capture correlations among hidden variables (e.g. different drivers will maintain roughly the same speed level).

**Fig 1:**
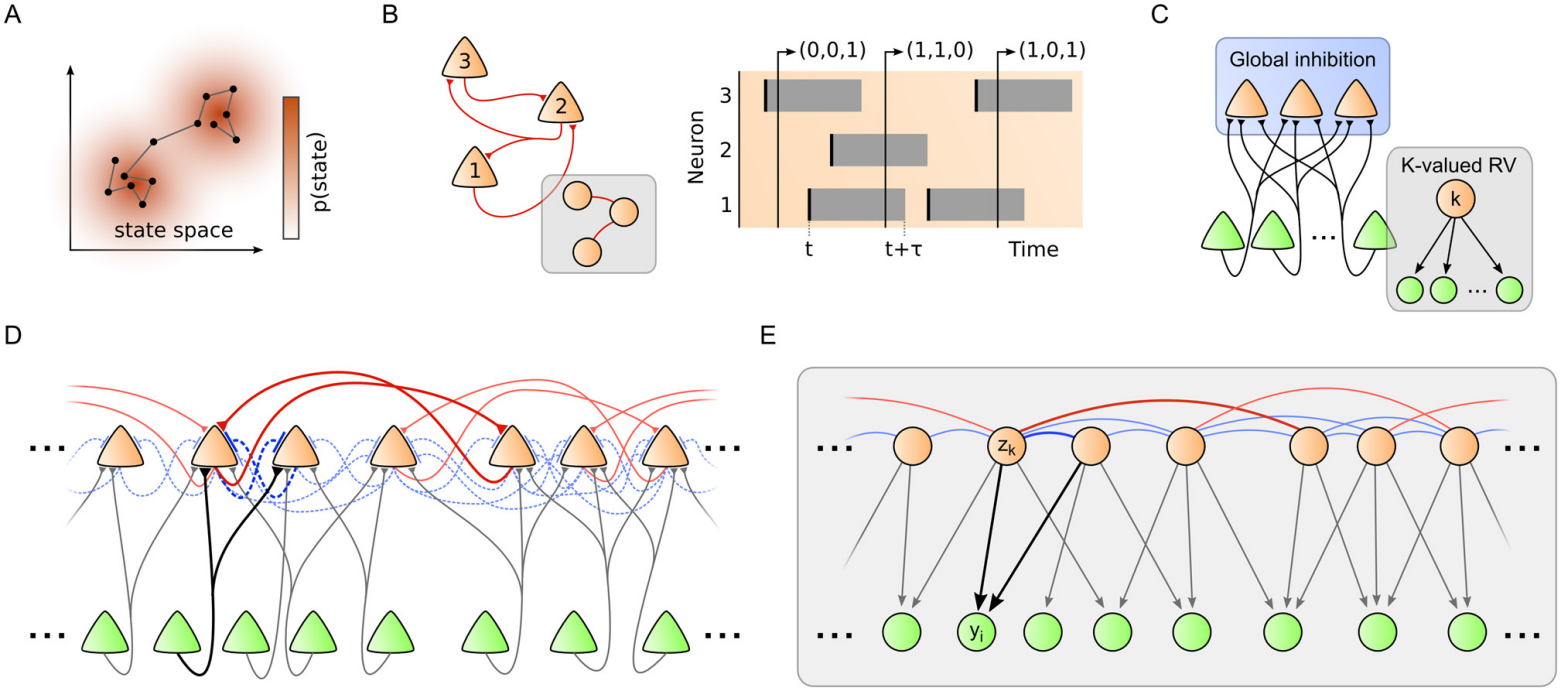
Neural sheet model with local inhibition for distributed Bayesian inference and self-organized learning. (A) The sampling hypothesis proposes that probability distributions are represented in the brain such that the time the network spends in state ***z*** is proportional to the probability *p*(***z***). (B) In [17] it was shown that recurrent networks of stochastic spiking neurons can implement Markov chain Monte Carlo sampling in a well-defined graphical model (inset). Each neuron is identified with a binary random variable (RV). The state of the RV at time *t* encodes whether the neuron has fired shortly before (right). (C) In [25] it was shown that a local population of neurons (orange), organized in a Winner-Take-All (WTA) architecture, can learn an implicit probabilistic model of spiking input (green) through STDP-type plasticity. In [25], competition between the neurons was established via a global inhibitory current. Inset: Corresponding graphical mixture model. (D) We propose a spatially structured neural sheet model with lateral inhibition and recurrent excitation for distributed Bayesian computation and self-organized learning. The network model unites the benefits of [17] and [25]. Strong inhibitory connections (dashed blue) between nearby network neurons establish local competition. Sparse recurrent excitatory synapses (red) connect more distant neurons. In addition, each network neuron integrates spiking input from a local subset of input neurons (green). (E) Graphical model of the neural sheet in D. Nearby binary network RVs ***z***_*k*_ (orange nodes) maintain competitive links (blue) while more distant variables can maintain associative links (red). Bottom-up input synapses in D give rise to generative downward arrows to the input RVs *y*_*i*_ (green nodes).

During recent years, several modeling approaches explored how Bayesian computations can be performed by spiking networks, and how the involved probability distributions can be represented by the neuronal spike response [10–22]. Two major lines of research regarding the neural representation of probability distributions can be distinguished: distributional (probabilistic) population codes [10, 13] and sample-based representations [23, 24]. In sample-based representations, spike responses are interpreted as samples ***z*** from the posterior distribution as illustrated in Fig 1A: Through its inherent dynamics, the network trajectory visits states ***z***(*t*) proportionally to *p* (***z*** ∣ ***y***, ***θ***). Thus, neural activity is hypothesized to encode distributions in the sequence of network states. Sample-based representations are particularly appealing for theoretical considerations, since they are highly versatile and naturally support the representation of complex, potentially multimodal distributions over large numbers of variables [18, 24].

Recently, a generic spiking network model that samples from a known probability distribution was proposed by Buesing et al. [17]. The underlying theory describes the dynamics of networks of idealized stochastic spiking neurons as a Markov chain Monte Carlo (MCMC) sampling algorithm. In this model, each binary RV *z*_*k*_ ∈ {0,1} is associated with one spiking neuron in the network, and spikes of these neurons are interpreted as realizations of the corresponding RVs, see Fig 1B. After a spike of the k-th neuron at time *t* ^*s*^, the associated RV *z*_*k*_ turns active, i.e. *z*_*k*_ = 1, for a fixed duration *τ*, typically on the order of 10 milliseconds. At time *t* ^*s*^ + *τ*, the RV switches back to the inactive ground state *z*_*k*_ = 0. This interpretation of neuronal spike patterns as realizations of RVs defines the vector ***z*** = (*z*_1_, .., *z*_*K*_) of all associated RVs at any time *t*:

$$z_{k}\left(t\right)=1\Leftrightarrow \text{Neuron k fired in}\;\left(t-\tau ,t\right].$$ (1)

Based on this link between random variables and neuronal spike responses, the authors of [17] identified a sufficient condition for a population of stochastic spiking neurons to sample from a well-defined probability distribution *p*(***z*** ∣ ***θ***), i.e., the relative occurrences of states ***z***(*t*) visited by the network trajectory are distributed according to *p*(***z*** ∣ ***θ***) in the limit *t* → ∞. This implementation of MCMC sampling in networks of spiking neurons was termed *neural sampling*. The inset of Fig 1B depicts the graphical model of the distribution *p*(***z*** ∣ ***θ***) which the small neural network in the figure samples from. Notably, the links in the graphical model mirror the structure of synaptic network connections.

The neural sampling theory explains how networks of spiking neurons can sample from a given distribution *p*(***z*** ∣ ***θ***) over latent variables ***z*** for given parameters ***θ***. It does, however, not cover the question how these latent variables enter the network in the first place, i.e., how observed input ***y*** can be integrated and represented through latent variables ***z*** and how this representation can be learned from the statistics of observables ***y***. This question is addressed in the current article. In particular, we exhibit a network architecture for sheets of stochastically spiking neurons for which

- the spike response can be understood as neural sampling from the Bayesian posterior distribution of a well-defined probabilistic model,
- local synaptic plasticity rules can be derived for self-organized model optimization of the parameters ***θ*** using machine learning theory, and
- emerging recurrent connections store correlations between latent variables ***z*** and help to maintain coherent network states for resolving ambiguous input.

For the self-organized adaptation of network parameters ***θ*** based on the statistics of observations ***y***, we adopt a “generative perspective” which is often used in Bayesian modeling [9, 25–27]. In the generative perspective, we interpret the latent variables ***z*** as so-called hidden causes of the inputs ***y***. This view permits to identify a conditional distribution *p* (***y*** ∣ ***z***, ***θ***) (called “likelihood”) which describes the distribution over inputs if the network would (hypothetically) generate its own spiking inputs based on its current network state ***z***. The conditional distribution *p* (***y*** ∣ ***z***, ***θ***) describes how likely an observation ***y*** is under the assumption that it was generated by the hidden cause ***z***. In a complementary, reversed perspective, by holding the observation ***y*** fixed and viewing *p* (***y*** ∣ ***z***, ***θ***) as a function of ***z***, this allows to assess how well different ***z***-configurations could serve as explanations for the given observation. This reversed perspective is formalized in Bayes rule

$$p\;(z\;|\;y,\;\theta )=\frac{p\;(y\;|\;z,\;\theta )\;p\;(z\;|\;\theta )}{p\;(y\;|\;\theta )}.$$ (2)

Bayes rule (2) tells us how to infer the statistically optimal posterior distribution *p* (***z*** ∣ ***y***, ***θ***) of network responses ***z*** to any stimulus ***y***. For this inference, two components are essential. The *prior* *p*(***z*** ∣ ***θ***) encodes that some combinations of latent variables are generally more likely than others (e.g. driving speeds of cars are highly correlated during rush hour). The *likelihood* *p* (***y*** ∣ ***z***, ***θ***) formalizes the constraint that the values of latent variables ***z*** should be such that the current observations ***y*** are probable under the generative probabilistic model. Note that the shape of both distributions is determined by the network parameters ***θ***. The denominator *p*(*y* ∣ ***θ***) can often be ignored since it just provides a normalizing factor for the posterior. A key benefit of the generative perspective is that it provides a theoretically well-founded approach to self-organized (unsupervised) learning. This is because inference of hidden causes works best when the parameters ***θ*** are tuned such that the hypothetical generative distribution of the network *p*(*y* ∣ ***θ***) matches the true distribution of observables p*(***y***). The process of minimizing the mismatch between the probabilistic model and the input statistics is known as “maximum likelihood learning”.

Adopting the generative perspective, Nessler et al. [25] recently developed a model for inference and learning in local populations of stochastic spiking neurons with lateral inhibition, see Fig 1C. Network neurons in this model receive all-to-all connections from a set of input neurons, and each network neuron maintains a set of afferent synaptic weights that render it an expert for detecting certain input patterns. A global inhibitory current enforces competition among the network neurons. Nessler et al. showed that the spiking activity of such a network with *K* neurons in response to input can be understood as Bayesian inference in an implicit probabilistic mixture model with *K* hidden causes (see inset of Fig 1C). Furthermore, it was shown that maximum likelihood learning in this model gives rise to synaptic update rules that appear compatible with experimental data on spike-timing dependent plasticity (STDP) [25, 28].

In this article, we combine the benefits of maximum likelihood learning in networks with lateral inhibition with the general theory of neural sampling. We consider a spatially structured network architecture (see Fig 1D) where network neurons represent latent variables ***z***. The spatially extended architecture generalizes the network motif considered in [25] in that network neurons inhibit each other locally through lateral inhibition [29] and, in addition, may form sparse excitatory connections beyond the range of lateral inhibition [30, 31]. Afferent connections from input neurons, that represent observables ***y***, branch and synapse locally in the sheet of network neurons. Lateral inhibition structures the network in local WTA-like subcircuits similar to [25] such that network neurons that receive input from overlapping sets of input neurons are subject to strong lateral inhibition. This constraint ensures that theoretically correct inference and learning can be implemented through simple local neural operations (see Results and Discussion). In contrast to [25] however, which considered only a single WTA circuit motif, each network neuron in the architecture proposed here can participate in several WTA-subcircuits, and multiple network neurons with disjoint input can be recruited in parallel to cooperatively explain the spatially distributed input. Building on the neural sampling theory [17], we show that the response of this network architecture to spiking input can be understood as neural sampling-based Bayesian inference in the structured graphical model shown in Fig 1E. The graphical model has two main components: recurrent links between network nodes, and generative input links pointing from network nodes to input nodes. The recurrent links encode statistical correlations between latent variables ***z*** by shaping the prior distribution *p*(***z*** ∣ ***θ***) in Bayes rule Eq (2). The generative input links encode the likelihood model *p* (***y*** ∣ ***z***, ***θ***). We show that both these components of the probabilistic model can be optimized concurrently through local synaptic plasticity rules in this network architecture. In particular, we derive iterative update rules for maximum likelihood learning which give rise to Hebbian-type synaptic and homeostatic intrinsic plasticity rules in the neural network. The joint application of these rules can be understood as Stochastic Online Expectation-Maximization [32], a powerful machine learning algorithm for unsupervised model optimization. While a theoretically optimal STDP-type plasticity rule can be derived for afferent connections that define the likelihood model *p* (***y*** ∣ ***z***, ***θ***), an approximate solution is proposed for recurrent connections that define the prior distribution *p*(***z*** ∣ ***θ***) over latent variables. In computer simulations, we verify that the spiking network can calculate and represent the theoretically correct Bayesian posterior distribution with high accuracy. We demonstrate how synaptic plasticity shapes the network response to extract and convey the most salient features of the input in a sparse distributed spike code, and how recurrent connections capture correlations between latent variables ***z*** to maintain a coherent network-wide interpretation. These simulations also reveal how the sampling network calculates and represents uncertainty in case of ambiguous or uninformative input. When the presented input appears consistent with multiple possible (but mutually incompatible) explanations, the network response encodes the associated multimodal posterior distribution *p* (***z*** ∣ ***y***, ***θ***) by switching iteratively between different coherent network states. Finally, our theoretical analysis points to an integral role of lateral inhibition during learning: it is the local inhibition network motif that gives rise to local synaptic plasticity rules and that facilitates the emergence of probabilistic local experts. The resulting well-adapted network transforms high-dimensional spiking input streams into an efficient sparse code.

The remainder of the manuscript is structured as follows. We first introduce the probabilistic model and show how the resulting posterior distribution *p* (***z*** ∣ ***y***, ***θ***) can be calculated and represented by a spiking neural network with local afferent connections, lateral inhibition and sparse recurrent excitation through neural sampling. We then investigate synaptic learning of afferent connections and recurrent connections in separate subsections. Finally, we apply the complete spiking network architecture to a two-dimensional model of neural tissue in which we observe the emergence of excitatory subnetworks through the interplay of afferent and recurrent synaptic plasticity.

## Results

### Probabilistic inference in spatially extended spiking networks

In this section, we derive how the spike response of the neural sheet model with the architecture in Fig 1D can be understood as an ongoing sampling process from a Bayesian posterior distribution *p* (***z*** ∣ ***y***, ***θ***) that arises from the graphical model in Fig 1E.

#### Spiking neural network model

The network architecture in Fig 1D comprises network neurons and input neurons. The spiking activity of input neurons is fed externally into the network. For the network neurons, we employ the stochastic spike response neuron model from Buesing et al. [17] that describes the state of each network neuron by a binary variable *z*_*k*_(*t*) ∈ {0,1} according to Eq (1): After a spike of the k-th network neuron, *z*_*k*_(*t*) turns active for duration *τ*. After that period, the variable switches back to the inactive state *z*_*k*_(*t*) = 0. Similarly, the state of the i-th input neuron is described by a binary variable *y*_*i*_(*t*) ∈ {0,1} with *y*_*i*_(*t*) = 1 for duration *τ* after a spike of the i-th input neuron. The membrane potential of the *k*-th network neuron is given by

$$u_{k}\left(t\right)=b_{k}+\overset{K}{\underset{j=1}{\sum }}W_{kj}^{\text{exc}}\;z_{j}\left(t\right)+\overset{K}{\underset{j=1}{\sum }}W_{kj}^{\text{inh}}\;z_{j}\left(t\right)+\overset{N}{\underset{i=1}{\sum }}V_{ki}\;y_{i}\left(t\right).$$ (3)

Here, *b*_*k*_ denotes a neuron’s intrinsic excitability and captures, for instance, the influence of the voltage gap between resting and threshold potential in more detailed neuron models [19, 33]. $W_{kj}^{\text{exc}}$ and $W_{kj}^{\text{inh}}$ are recurrent synaptic weights between network neurons to instantiate sparse recurrent excitation [30] and local lateral inhibition [34]. For simplicity, we model the effect of inhibition as direct negative connections $W_{kj}^{\text{inh}}$ between network neurons, i.e., we do not model interneurons explicitly. The afferent weights *V*_*ki*_ denote the strength of synapses from the i-th input to the k-th network neuron. For notational convenience, weight 0 is assigned to non-existing connections. As in [17], neurons communicate via rectangular post-synaptic potentials (PSPs) of duration *τ* and with amplitude $W_{kj}^{\text{exc}}$, $W_{kj}^{\text{inh}}$ and *V*_*ki*_ respectively. Throughout this work, we chose *τ* = 10 *ms* as an estimate for PSP durations. Thus, the membrane potential *u*_*k*_(*t*) integrates the current value of all presynaptic variables *z*_*j*_(*t*) and *y*_*i*_(*t*) at any time.

Network neurons emit spikes stochastically with instantaneous firing probability

$$\rho_{k}\left(t\right)=\underset{\delta t\to 0}{\text{lim}\;}\frac{1}{\delta t}p\left(\text{Neuron k fires in}\;\left[t+\delta t\right)\right)=\frac{1}{\tau }e^{u_{k}\left(t\right)}\left(1-z_{k}\left(t\right)\right).$$ (4)

Here, (1 − *z*_*k*_(*t*)) describes a refractory period that is inversely related to the state *z*_*k*_(*t*), i.e., when *z*_*k*_(*t*) is active, the neuron is refractory and cannot emit another spike. The exponential dependence of the firing probability *ρ*_*k*_ on *u*_*k*_ was confirmed to be a reasonable modeling assumption for neurons in a noisy environment [19, 33].

#### The network response as the result of a meaningful Bayesian computation

We aim to understand the activity of the network neurons in response to spiking input as the result of a Bayesian computation. To establish a link between the stochastic spike response properties of individual neurons and the joint activity distribution of the entire network, we build upon the neural sampling theory [17] where states *z*_*k*_(*t*) and *y*_*i*_(*t*) of the neurons are formally treated as random variables *z*_*k*_ and *y*_*i*_. This probabilistic description of stochastic network activity in response to given input amounts to a conditional probability distribution *p* (***z*** ∣ ***y***, ***θ***) for each possible input configuration ***y***. In order to assign a computational meaning to the conditional distributions *p* (***z*** ∣ ***y***, ***θ***), we will identify a generative model, consisting of prior *p*(***z*** ∣ ***θ***) and likelihood *p* (***y*** ∣ ***z***, ***θ***), for which the conditional distributions *p* (***z*** ∣ ***y***, ***θ***) arise from Bayes rule (2) as the correct posterior. The network’s spike response can then be understood as probabilistic inference through sampling from the posterior *p* (***z*** ∣ ***y***, ***θ***).

To decide whether the posterior distribution of a generative model is compatible with the neural dynamics of the spiking network, we make use of a sufficient condition, called *neural computability condition* [17], which connects the membrane potentials *u*_*k*_ of individual spike response neurons (4) with the activity distribution of the recurrent network. For our case of a posterior distribution *p* (***z*** ∣ ***y***, ***θ***) with arbitrary (but fixed) input ***y***, the neural computability condition reads:

$$u_{k}\;\overset{!}{=}\;\text{log}\frac{p\;\left(z_{k}=1|z_{\backslash k},\;y,\;\theta \right)}{p\;\left(z_{k}=0|z_{\backslash k},\;y,\;\theta \right)}\;\text{for}\;\text{all}\;k=1,..,K$$ (5)

with ***z***_\*k*_ = (*z*_1_, .., *z*_*k*−1_, *z*_*k* + 1_, .., *z*_*K*_) denoting the state of the remaining network. If the condition holds for all possible input states ***y*** and all possible network states ***z***, the trajectory of network states ***z***(*t*) is guaranteed to be distributed according to *p* (***z*** ∣ ***y***, ***θ***) in the limit *t* → ∞ for any fixed input ***y***, i.e., the spiking network samples from *p* (***z*** ∣ ***y***, ***θ***).

To establish the equivalence between the spiking network in Fig 1D and the Bayesian model in Fig 1E, we introduce prior *p*(***z*** ∣ ***θ***) and likelihood distributions *p* (***y*** ∣ ***z***, ***θ***) which are shaped by parameters ***θ*** and match the graphical model in Fig 1E. From these distributions, we then calculate the posterior *p* (***z*** ∣ ***y***, ***θ***) according to Bayes rule (2). Finally, we apply the sufficient condition (5) to decide under what conditions the spiking network will sample from the correct posterior. In particular, this will allow us to determine the connectivity structure of the network in detail, and furthermore, to precisely map the parameters of the generative model to the synaptic efficacies and intrinsic excitabilities of the neural sheet model.

We set out with a class of prior distributions *p*(***z*** ∣ ***θ***), namely Boltzmann distributions, that have been shown [17] to be compatible with the neural sampling dynamics of the recurrent spiking network model (without input):

$$p\;(z\;|\;\theta )=\frac{1}{Z}\;\exp[\frac{1}{2}z^{⊺}{\hat{W}}^{\text{exc}}z+\frac{1}{2}z^{⊺}{\hat{W}}^{\text{inh}}z+z^{⊺}\hat{b}],$$ (6)

where *Z* is a normalizing factor. The distribution assigns probabilities to binary random vectors ***z*** = (*z*_1_, .., *z*_*K*_)^⊺^ with *z_k_* {0,1}. The parameters $\hat{b},\;{\hat{W}}^{\text{exc}},\;{\hat{W}}^{\text{inh}}$ shape the distribution and consist of a real-valued bias vector $\hat{b}={\left({\hat{b}}_{1},\;\ldots ,{\hat{b}}_{K}\right)}^{⊺}$ and symmetric, zero-diagonal *K* × *K* coupling matrices ${\hat{W}}^{\text{exc}}$ and ${\hat{W}}^{\text{inh}}$. We endow the prior (6) with the spatial structure sketched in the upper row of Fig 1E. Orange circles depict the random variables *z*_*k*_ that correspond to network neurons. The random variables *z*_*k*_ maintain sparse excitatory recurrent connections ${\hat{W}}_{kj}^{\text{exc}}$ (red links) on an intermediate range. Excitatory links between variables make their coactivation more probable in the prior (6) and thus encode network-wide state combinations ***z*** that are more likely to occur than others. This associative memory aspect will turn out to be particularly powerful in the context of recurrent learning where structural knowledge is integrated by the prior and will allow the network to maintain coherent network states even in face of ambiguous input. The sparse excitatory links on intermediate distances are complemented with strong negative connections ${\hat{W}}_{kj}^{\text{inh}}$ (theoretically ${\hat{W}}_{kj}^{\text{exc}}\to -\infty$, blue links) on a local scale among nearby network neurons. These negative connections will correspond to local lateral inhibition in the network and, as we will see in the context of learning, facilitate self-organized statistical model optimization through synaptic plasticity.

The likelihood distribution *p* (***y*** ∣ ***z***, ***θ***) establishes the connection between the network variables *z*_*k*_ and the input variables *y*_*i*_. We adopt a generative perspective in order to identify the likelihood distribution *p* (***y*** ∣ ***z***, ***θ***) and view the network variables *z*_*k*_ as “hidden causes” of their inputs *y*_*i*_. Thus, we assume that each input *y*_*i*_ is (hypothetically) generated by the corresponding subset of connected network variables *z*_*k*_. This amounts to the downward arrows in Fig 1E where each input *y*_*i*_ (green circles) receives converging arrows only from nearby network variables ***z***_*k*_, the so-called parents of *y*_*i*_. For our idealized architecture, we further assume that no two parents *z*_*k*_ and *z*_*j*_ of an individual input *y*_*i*_ can be active simultaneously. This is ensured by ***z***_*k*_ and ***z***_*j*_ sharing strong negative connections ${\hat{W}}_{kj}^{\text{inh}}$, i.e., the parents of *y*_*i*_ are assumed to lie within the range of lateral inhibition (blue links in Fig 1E). As a consequence, each input *y*_*i*_ is generated by at most one of its parents at a time. We denote the probability of *y*_*i*_ to be active by *π*_*ki*_ (with 0 < *π*_*ki*_ < 1) given that it is generated by the active hidden cause *z*_*k*_. If none of its parents is active, the chance of activity is assigned a constant default value *π*_0*i*_. This is summarized in the following Bernoulli distribution:

$$p\left(y_{i}=1|z,\theta \right)=\{\begin{array}{cc} \pi_{ki} & \text{ if}z_{k}=1\text{ for a parent }z_{k}\text{of }y_{i}\\ \pi_{0i} & \text{ if}z_{k}=0\text{ for all parents of }y_{i}. \end{array}$$ (7)

To obtain the full likelihood distribution *p* (***y*** ∣ ***z***, ***θ***) over all *N* input variables, we note that the *y*_*i*_’s are conditionally independent and, hence, $p_{}\left(y\;|\;z,\;\theta \right)=\prod_{i=1}^{N}p_{}\left(y_{i}\;|\;z,\;\theta \right)$. This completes the definition of prior and likelihood distributions that match the graphical model sketched in Fig 1E. A formal definition of the probabilistic model is provided in *Generative model* in Methods. There we also describe extensions of the likelihood distribution to support natural and real-valued inputs, i.e., to *y*_*i*_ ∈ ℕ and *y*_*i*_ ∈ ℝ.

#### The neural network can sample from the posterior of the generative model

From the prior (6) and the likelihoods (7), the posterior distribution *p* (***z*** ∣ ***y***, ***θ***) can be calculated in closed-form using Bayes rule. By applying the neural computability condition (5), a straightforward derivation reveals that the membrane potential (3) is compatible with the posterior *p* (***z*** ∣ ***y***, ***θ***) of the generative model. The closed-form posterior and the derivation are provided in *Inference in the generative model* in Methods. Furthermore, the calculation yields a translation between the network parameters $b_{k},W_{kj}^{\text{exc}},W_{kj}^{\text{inh}},V_{ki}$ and the abstract parameters ${\hat{b}}_{k},{\hat{W}}_{kj}^{\text{exc}},{\hat{W}}_{kj}^{\text{inh}},\pi_{ki}$ (and the constants *π*_0*i*_), and results in the following corollary:

#### Corollary 1 (Inference)

*Let network parameters and abstract parameters be identified via*

$$\;b_{k}={\hat{b}}_{k}-A_{k}$$ (8)

$$V_{ki}=\text{log}\;(\frac{\pi_{ki}}{1-\pi_{ki}})-\text{log}\;\left(\frac{\pi_{0i}}{1-\pi_{0i}}\right)$$ (9)

$$W_{kj}^{\text{exc}}={\hat{W}}_{kj}^{\text{exc}},\;W_{kj}^{\text{inh}}={\hat{W}}_{kj}^{\text{inh}}$$ (10)

*with* $A_{k}≔\sum_{i=1}^{N}\text{log}\;\left[1+\pi_{0i}\cdot \left(e^{V_{ki}}-1\right)\right]$, *and let the recurrent weights* ${\hat{W}}^{\text{exc}}$ *and* ${\hat{W}}^{\text{inh}}$ *be symmetric matrices with zero diagonal. Then, for any fixed input instantiation **y**(t) = **y**, the response **z**(t) of a recurrent spiking network consisting of stochastic neurons* (4) *with linear membrane potential* (3) *is distributed according to*

z(t)∼p(z|y,θ)

*in the limit t → ∞, i.e. the network samples from the posterior distribution of the probabilistic model defined by the prior* (6) *and the likelihoods* (7).

Corollary 1 explains the structural similarity of the graphical model in Fig 1E and the network architecture in Fig 1D: Each red (blue) recurrent link between latent variables *z*_*k*_ and *z*_*j*_ in the graph corresponds to a symmetric reciprocal excitatory (inhibitory) synaptic connection between the k-th and j-th network neuron; and each downward arrow from *z*_*k*_ to *y*_*i*_ in the graph gives rise to a synapse *V*_*ki*_ from the i-th input neuron to the k-th network neuron. In particular, the assumption in the abstract model, that at most one parent *z*_*k*_ explains a dependent input variable *y*_*i*_ at a time, translates to a local lateral inhibition motif in the spiking network: Any two network neurons, that share common input, inhibit each other through lateral inhibition. This theoretically derived network motif is reminiscent of cortical lateral inhibition frequently reported across areas and species [29]. In the neural sheet model, local lateral inhibition introduces Winner-take-all (WTA) competition among nearby network neurons which thus play the role of local feature detectors. However, the local WTA circuits are not separated in the sheet, but rather interwoven such that each network neuron can participate in multiple overlapping WTA sub-circuits. In addition, lateral excitatory connections $W_{kj}^{\text{exc}}$ encode associations between spatially distant feature combinations. This generalized concept of WTA circuits in the continuous sheet contrasts with existing models of interacting WTAs [35] which investigated disjoint non-overlapping WTA sub-circuits that operate in parallel. The full probabilistic model *p*(***y***, ***z*** ∣ ***θ***) of the neural sheet can thus be understood as a spatially extended reservoir of contiguous competing local feature detectors (corresponding to the likelihood *p* (***y*** ∣ ***z***, ***θ***)) and an associative memory over the feature set (corresponding to the prior *p*(***z*** ∣ ***θ***)).

**Fig 2:**
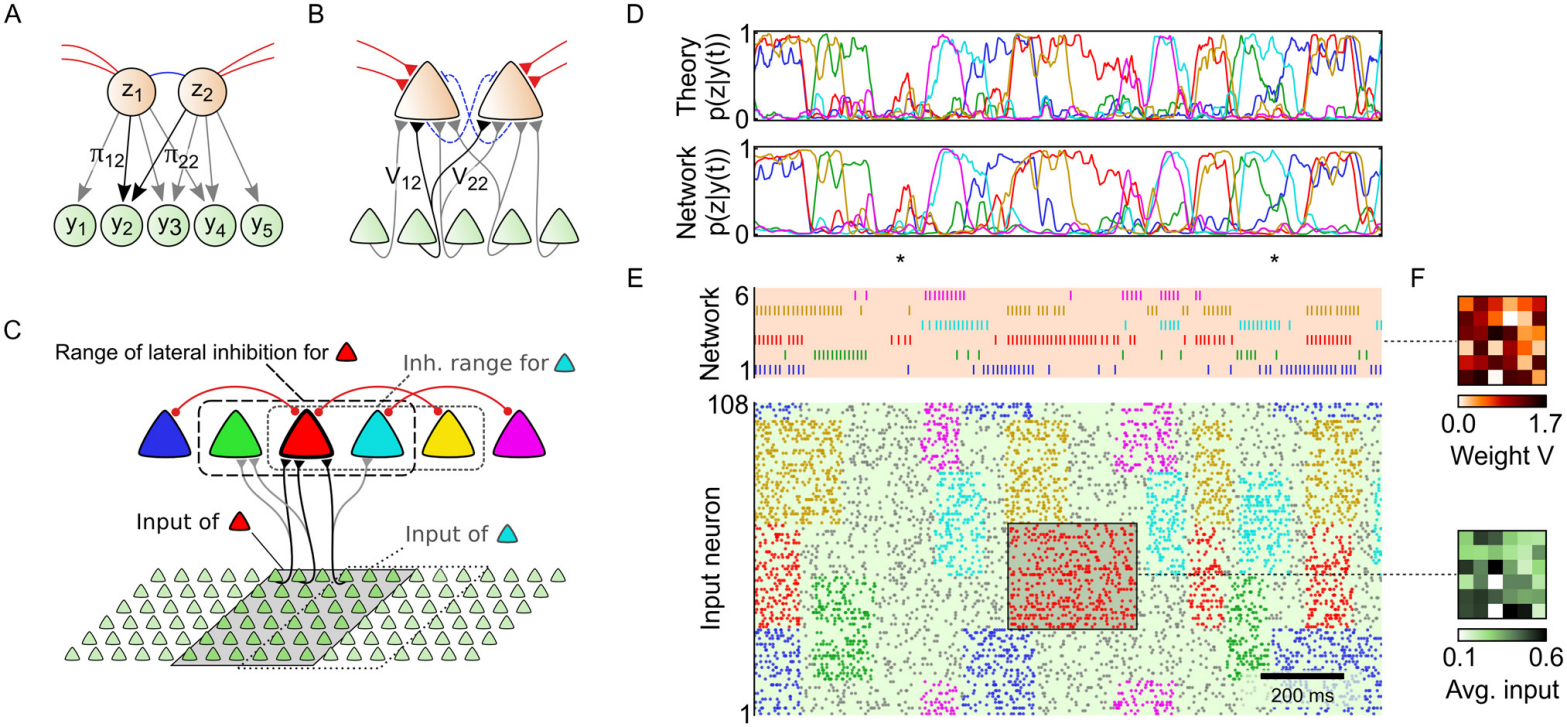
Sheets of spiking neurons can perform Bayesian inference on distributed spiking input. (A) Local generative model with two competing hidden causes and five inputs. Each hidden cause stores a specific input pattern in the top-down parameters *π*_*ki*_. (B) Corresponding local neural network. Top-down parameters *π*_*ki*_ translate to bottom-up synaptic weights *V*_*ki*_, turning each network neuron into a probabilistic expert for a specific local input pattern. (C) Example network with six network neurons. Neighboring neurons with overlapping input inhibit each other (dashed line: range of lateral inhibition for red neuron). The spiking network is linked to a generative model *p* (***y***, ***z*** ∣ ***θ***) according to Corollary 1. (D) The network in C performs sample-based inference of hidden causes ***z*** under time-varying input ***y***(*t*) in the associated generative model. Comparison of posterior marginals *p* (*z*_*k*_ = 1 ∣ ***y***(*t*), ***θ***) between the analytically calculated exact posterior (top) and the average network response (bottom, estimated from 1000 simulation runs) under the time-varying input ***y***(*t*) in panel E. All traces were smoothed with a 20ms box kernel for visual clarity. (E) Top: Network spike response in a single simulation run. Bottom: Input spike trains (colored: structured input, gray: background activity). The network response is an ongoing sampling process from the posterior *p* (***z*** ∣ ***y***(*t*), ***θ***). Time points marked by a ‘*’ exemplify characteristic properties of sample-based Bayesian information processing. (F) Top: Bottom-up weights *V*_*ki*_ of the red neuron. Bottom: Average input activity while the matching pattern is presented (during the 300ms period marked in E).

The implementation of Bayes rule (2) by the spiking network is illustrated in Fig 2A and 2B for the minimal example of two neighboring neurons with an overlapping, but not identical, subset of inputs *y*_*i*_. Both neurons *z*_1_ and *z*_2_ maintain an implicit likelihood model for their respective local inputs (top-down arrows in A, bottom-up synapses in B). Additionally, the prior installs competition between the neurons via negative reciprocal connections *W* ^inh^ (blue edge in A, blue synapses in B). Consequently, the neurons *z*_1_ and *z*_2_ preferentially fire to different input patterns ***y***. Corollary 1 guarantees that the frequency of occurrence of each network state (*z*_1_, *z*_2_) will be proportional to *p* (*z*_1_, *z*_2_ ∣ ***y***, ***θ***) for any input instantiation ***y***. This property is particularly important for inputs ***y*** that are roughly equally compatible with the preferred activity patterns of both network neurons. In such ambiguous cases, the network trajectory will sample the posterior states (*z*_1_, *z*_2_) = (0,0), (0,1) and (1,0) in accordance with Bayes rule. As a consequence, the network response carries information on not only the most likely solution but also on the (un-)certainty of all possible outcomes—a pivotal aspect of Bayesian information processing.

#### Demonstration of sample-based inference on transient spiking input

Corollary 1 ensures that the network will sample from the correct posterior distribution *p* (***z*** ∣ ***y***, ***θ***) in arbitrarily large network architectures for any fixed input configuration ***y***(*t*) = ***y***. However, Corollary 1 offers no strict guarantees in case of time-varying input since it only ensures that the network activity converges to the equilibrium distribution *p* (***z*** ∣ ***y***, ***θ***) in the limit *t* → ∞. For transient spiking input, the underlying Markov chain cannot fully converge to its equilibrium distribution and the sampling network will “lag behind” the transient posterior *p* (***z*** ∣ ***y***(*t*), ***θ***).

We therefore assessed the network’s ability to sample from the analytically correct posterior distribution in case of time-varying input ***y***(*t*) in a computer simulation. The computer simulation was performed in the setup of the small, analytically tractable network architecture shown in Fig 2C. This 6-neuron network will furthermore serve to illustrate salient response properties of the neural sheet during neural sampling-based inference. The prior features both sparse excitatory connections and local lateral inhibition such that neighboring neurons compete with each other through inhibitory connections. More distant neurons maintain excitatory connections (red links: $W_{kj}^{\text{exc}}=1$), representing knowledge that these two hidden causes are likely to co-occur. Furthermore, each neuron has a (randomly generated) preferred local input pattern stored in its synaptic weights *V*_*ki*_. For instance, the top panel of Fig 2F shows the afferent weights of the red neuron. A demonstration of neural sampling by this circuit in response to time-varying input ***y***(*t*) is shown in Fig 2E: High-dimensional (*N* = 108) input spike trains are presented to the network (bottom). Input spikes consist of uninformative background spikes (gray), interleaved with periods of more structured inputs (colored spikes). Uninformative background spikes are Poisson spike trains with a uniform rate chosen such that the average activation of input neurons is 〈 *y*_*i*_ 〉 = 0.2 (ca. [20]*Hz*). Structured input spikes are Poisson spike trains with rates that were chosen to match the activity patterns stored in the afferent weights *V*_*ki*_ of the network neurons. For example, the red neuron is particularly good at detecting the red spike pattern. Fig 2E (top) shows the network response to the input spike pattern (bottom) during a single simulation run. Whenever a structured local input pattern occurs, the corresponding network neuron starts firing. Yet, the network response is not deterministic: sometimes network neurons elicit spikes even when presented with unstructured input (see e.g. at the 1^st^ time point marked by a ‘*’); in other cases, a competing neighboring neuron emits a spike (see e.g. at the 2^nd^ time point marked by a ‘*’). Due to the stochastic nature of the network response, the exact spike pattern of the network will be slightly different in each simulation run. This apparent trial-to-trial variability is an inherent feature of sample-based representations.

We next turn to the question how well the network trajectory ***z***(*t*) approximates the correct Bayesian posterior *p* (***z*** ∣ ***y***(*t*), ***θ***) at any time *t*. A quantitative comparison addressing this question is provided in Fig 2D. From many repetitions of the experiment—all with the same input spike pattern ***y***(*t*)—we can estimate the distribution the network actually samples from. The top row of Fig 2D shows the exact marginal probabilities *p*_theo_ (*z*_*k*_ = 1 ∣ ***y***(*t*), ***θ***) at any time *t*, analytically calculated from Bayes rule. The bottom row shows the average network response *p*_net_ (*z*_*k*_ = 1 ∣ ***y***(*t*), ***θ***), estimated from the samples from 1000 repetitions of the experiment. The comparison indicates that the sampling network approximates the correct posterior probabilities with high accuracy at almost any time, capturing not only qualitative aspects of the transient posterior distribution but also the quantitative composition of the distribution in face of input fluctuations. Only for particularly rapid input fluctuations, which lead to sharp peaks in the posterior, the network shows a slightly delayed and sometimes inaccurate response. A more detailed statistical evaluation of the sampling quality is provided in *Details to the computer simulations* in Methods, along with a brief discussion on the origins of stochastic and systematic deviations in the sampled distribution. In conclusion, the quantitative comparison in Fig 2D shows that the stochastic network response ***z***(*t*) can be understood as an ongoing Bayesian inference process, even in case of time-varying input ***y***(*t*).

Fig 2D and 2E also exemplify characteristic properties of sample-based Bayesian information processing. At times, input instantiations ***y***(*t*) may be noisy or ambiguous such that the hidden causes of the presented input cannot be inferred with certainty. Two typical examples are marked with a ‘*’ symbol: At the first time point marked, the unstructured background input accidentally bears some resemblance to the red and the yellow input patterns, such that the posterior probability for inferring the red/yellow patterns temporarily jumps to *p* (*z*_*k*_ = 1 ∣ ***y***(*t*), ***θ***) ≈ 1/2 for *k* = 3 and *k* = 5. This brief moment of uncertainty due to stochastic fluctuations in the input is represented by the network via a small number of spikes of the red (k = 3) and the yellow (k = 5) network neurons. While the exact timing of the spikes is a stochastic process, the instantaneous spiking probability in the sampling network is well in line with the analytically calculated posterior. At the second time point marked, two competing neurons (dark blue and green) could explain their local input well, as can be seen from the marginal posterior *p* (*z*_*k*_ = 1 ∣ ***y***, ***θ***). In such ambiguous situations, both neurons are eager to fire, yet they compete due to lateral inhibition. As a result, network activity switches between two local interpretations where either one of the two hidden causes is active. Due to the stochastic nature of the sampling process, the particular switching times between the competing network states change with each repetition of the experiment, leading to trial-to-trial variability from the perspective of an external observer.

### Emergence of local experts through synaptic plasticity

Animals and humans possess the ability not only to infer hidden causes of their perceptions, but also to adapt their internal model, that underlies these inferences, to their specific environment. From a Bayesian perspective, this amounts to reshaping the parameters of the internal model *p* (***y***, ***z*** ∣ ***θ***) to better suit the true input statistics. For our analysis of plasticity, we keep the lateral inhibition structure, i.e, the effect of interneurons, fixed, and focus on plasticity of excitatory synapses. The guiding questions for the following two subsections are:

- How can the afferent weights *V*_*ki*_, recurrent weights $W_{kj}^{\text{exc}}$ and neuronal excitabilities *b*_*k*_ be adapted to support inference in a statistically optimal manner?
- To what extent can this network-wide optimization be accomplished with only local plasticity rules?

To address these questions we employed a standard objective function [9] for statistical model optimization, namely the log-likelihood of the input under the model:

$$ℒ\left(\theta \right)={\langle \text{log}\;p\;(y\;|\;\theta )\rangle }_{p^{*}\left(y\right)},$$ (11)

where p*(***y***) denotes the true distribution of inputs ***y*** actually presented to the network. Eq (11) makes use of a conceptual advantage of the Bayesian approach: So far we were only interested in the posterior distribution *p* (***z*** ∣ ***y***, ***θ***) which describes the stochastic network response ***z*** under a given stimulus *y*; now we switch to the complementary generative view and examine the distribution *p* (***y*** ∣ ***θ***) = ∑_***z***_ *p* (***y***, ***z*** ∣ ***θ***) of data hypothetically generated by the model. The distribution *p*(***y*** ∣ ***θ***) can be viewed as the outcome when the probabilistic model would “dream” its own environment. Adopting the complementary view is only possible because a full probabilistic model *p* (***y***, ***z*** ∣ ***θ***) of the network is available. The function ℒ(***θ***) then measures the likelihood of the actually presented input ***y*** ∼ p*(***y***) to occur in *p* (***y*** ∣ ***θ***). Since ℒ(***θ***) = −*D*_KL_(p* ∣∣ *p*) + *const*., increasing ℒ(***θ***) is equivalent to reducing the Kullback-Leibler divergence *D*_KL_(p* ∣∣ *p*) that measures the dissimilarity between the two distributions. Therefore, maximizing ℒ(***θ***) means to align the internal model *p* (***y*** ∣ ***θ***) with the true input distribution p*(***y***). This objective is commonly known as “maximum likelihood learning” in the machine learning literature [9].

In a first step, we investigated the maximization of ℒ(***θ***) with respect to ***V***, i.e., we examined the role of plastic afferent synapses *V*_*ki*_. Plastic recurrent connections $W_{kj}^{\text{exc}}$ will be addressed in a separate subsection, and we set ***W*** ^exc^ = **0** for now. Update rules for the afferent weights *V*_*ki*_ can be directly derived from the probabilistic model using the mathematical framework of generalized online Expectation Maximization [25, 36]. The derivation is provided in *Model optimization via Generalized Expectation Maximization* in Methods, and yields the following plasticity rules for synaptic weights *V*_*ki*_ and intrinsic excitabilities *b*_*k*_:

$$\frac{\partial }{\partial t}\;V_{ki}=\eta_{V}\cdot z_{k}\left(t\right)\cdot (y_{i}\left(t\right)-\sigma \left(V_{ki}+V_{0i}\right))$$ (12)

$$\frac{\partial }{\partial t}\;b_{k}=\eta_{b}\cdot \left(m_{k}-z_{k}\left(t\right)\right).$$ (13)

Here, *η*_*V*_ and *η*_*b*_ denote small learning rates, *σ*(*x*) ≔ 1/(1 + exp(−*x*)) is the logistic function, *V*_0*i*_ ≔ log(*π*_0*i*_/(1−*π*_0*i*_)) is a constant in order to respect the default activity *π*_0*i*_, and *m*_*k*_ is a long-term average target response 〈*z*_*k*_〉 of the *k*-th network neuron. Importantly, both plasticity rules only rely on information that is locally and instantaneously available to the neurons and synapses: Each afferent synapse *V*_*ki*_ adapts its weight in a Hebbian-type update based on pre-(*y*_*i*_) and post-(*z*_*k*_) synaptic activity through the weight-dependent plasticity rule (12); each neuron changes its intrinsic excitability *b*_*k*_ in a homeostatic fashion based on its current spike response *z*_*k*_. The joint application of the rules (12) and (13) can be shown to implement unsupervised statistical model optimization in the sense of the following corollary:

#### Corollary 2 (Learning of afferent synapses)

*The implicit probabilistic model defined by* (6) *and* (7) *can be optimized with respect to the afferent synaptic parameters V_ki_ by a Generalized Expectation Maximization algorithm which continuously increases a lower bound*

𝓕≤ℒ(θ)

*of the log-likelihood ℒ(**θ**) until a local optimum of ℱ is reached. The concurrent application of the learning rules* (12) *and* (13) *implements an online approximation of this optimization algorithm.*

The approximate character of the network implementation only arises from incomplete convergence of the network’s Markov chain during inference and from the non-infinitesimally small learning rates *η*_*V*_ and *η*_*b*_. Ignoring these effects, i.e., in the limit of small learning rates (*η*_*b*_ → 0 and *η*_*V*_/*η*_*b*_ → 0) and assuming instantaneous convergence of the Markov chain, we find that the plasticity rules become exact. Then, the direction of the expected learning update is given by,

$${\langle z_{k}\cdot \left(y_{i}-\sigma \left(V_{ki}+V_{0i}\right)\right)\rangle }_{}=\frac{\partial 𝓕}{\partial V_{ki}}\;\text{for all}\;k,\;i,$$ (14)

where the expectation 〈⋅〉 is taken with respect to the presented input ***y***(*t*) and the network response ***z***(*t*). In other words, plastic changes in the synaptic weights *V*_*ki*_ point on average in the direction of increasing ℱ. Maximizing a lower bound ℱ on ℒ, instead of direct optimization of ℒ, is a common trick [37] in machine learning to obtain a tractable learning problem. In our model this approach serves to obtain a spiking network implementation of maximum likelihood learning in which all required information is available locally at the neurons and synapses. The local availability of information during learning comes at a cost. The network does not sample from the exact posterior anymore, but from a well-defined variational posterior [36]: The variational posterior, the network samples from, is the closest distribution to the analytically exact posterior (measured in terms of the Kullback-Leibler divergence) that satisfies the homeostatic long-term target activations *m*_*k*_. A full derivation of the plasticity rules (12) and (13), including a precise definition of the lower bound ℱ and the variational posterior distribution, is provided in *Model optimization via Generalized Expectation Maximization* in Methods.

#### Demonstration of self-organized learning in the neural sheet

**Fig 3:**
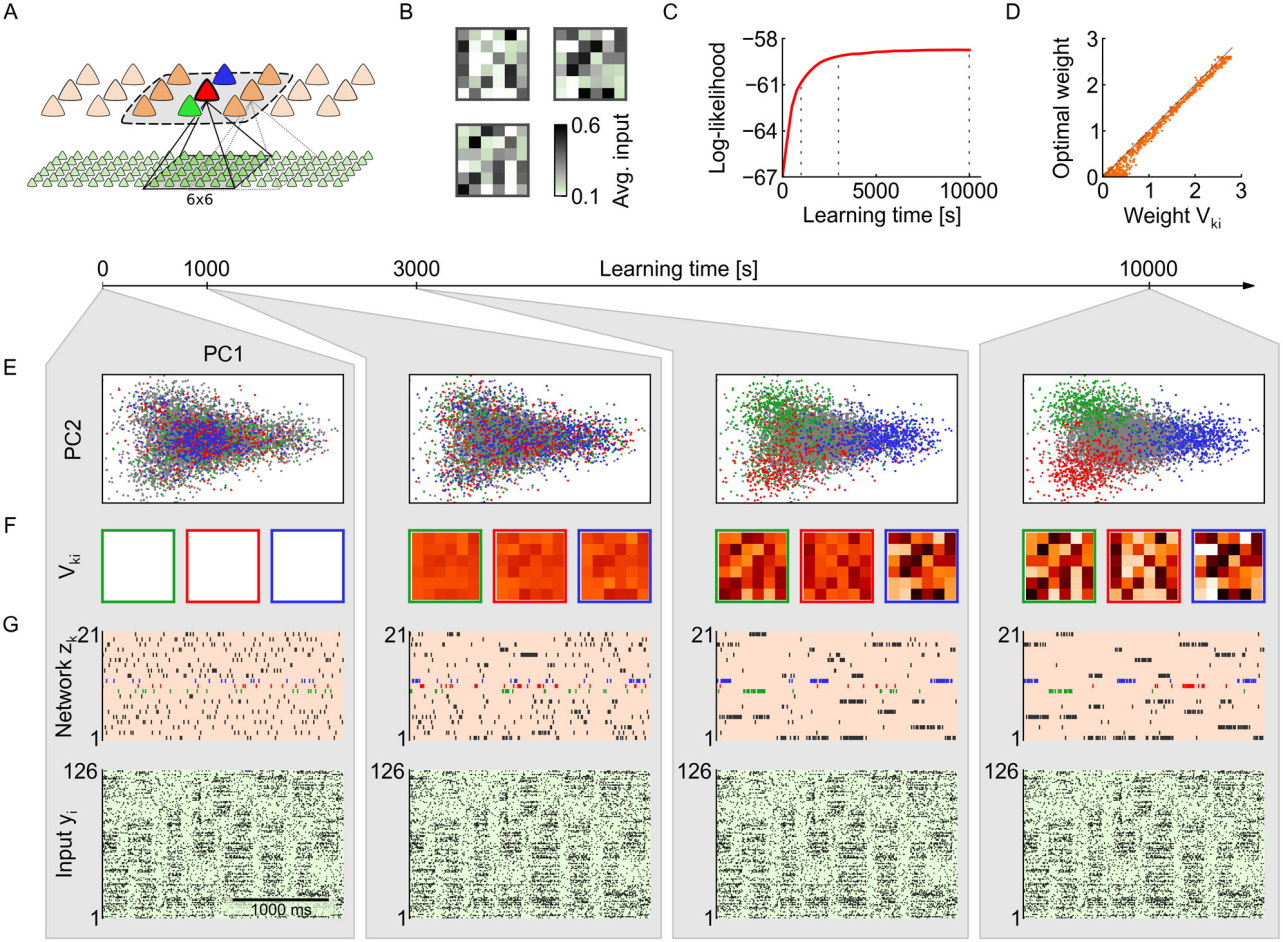
Emergence of probabilistic local experts through synaptic plasticity. (A) Network architecture with 21×6 inputs and 7×3 network neurons. The green, red and blue neuron receive input from the same 6×6 subset of input neurons. The input subsets of neighboring groups are shifted by three. The dashed line indicates the range of lateral inhibition. (B) At each of the overlapping 6×6 locations, three randomly drawn activity patterns can occur. Shown are the activity patterns for the location highlighted in A. (C) Synaptic and intrinsic plasticity shape the probabilistic model that is encoded by the network. The log-likelihood function **ℒ**(***θ***) measures how well the network is adapted to the presented input. (D) One-to-one comparison of the synaptic weights *V*_*ki*_ to analytically calculated optimal weight values, at the end of learning (*T* = [10, 000] *s*). (E) 2-dimensional projection of the local input distribution p*(***y***). Each dot is one input instantiation ***y***(*t*) at the 6×6 input field in A. Dots are colored according to which of the three neurons (green/red/blue) fired in response (gray: none fired). The neural plasticity rules achieve a clustering of local inputs into local categories. (F) Evolution of synaptic weights *V*_*ki*_ of the green/red/blue neuron over the course of learning. Each neuron becomes a probabilistic local expert for a certain input pattern (cp. panel B). (G) The plastic network develops a sparse, structured spike code that conveys compressed information about the presented input. Bottom: input spike trains. Top: Network response at different stages of learning.

We tested the derived learning rules for afferent weights *V*_*ki*_ and intrinsic excitabilities *b*_*k*_ in a computer simulation of a sheet of 7×3 network neurons (Fig 3A). The sheet model receives synaptic input from a total 21×6 afferent cells. Each network neuron receives input from a subset of 6×6 inputs. The network neurons are arranged such that in each column there are three neurons sharing the same 6×6 input (such as the green/red/blue neuron in Fig 3A). Neighboring columns of network neurons receive inputs from an overlapping subset of afferent cells (6×6 input subset shifted by three columns of input neurons to the left and right respectively). Neurons in the same column as well as neurons in neighboring columns inhibit each other. The input data was generated in a similar manner as in Fig 2, by interleaving a background Poisson spike train with periods of structured Poisson spikes generated from a small number of stereotypical rate patterns. At each 6×6 input field there are three such recurring activity patterns. At any moment, at most one such pattern is presented at any spatial position in the input. The resulting spike trains have complex spatio-temporal structure, as shown in Fig 3G (bottom).

Initially all afferent weights are set to *V*_*ki*_ = 0. Afferent weights are plastic and follow Eq (12). For the purpose of neuroscientific modeling, synaptic weights *V*_*ki*_ were restricted to positive values. The theory for inference and learning would support positive and negative weights, including sign changes. Excitabilities are uniformly initialized at *b*_*k*_ = −2 and follow Eq (13) with the average target response 〈*z*_*k*_〉 of each network neuron set to *m*_*k*_ = 6.5%. Whenever a network neuron spikes, synaptic plasticity is triggered and the current activity of the local 6×6 input field, that is connected to the active network neuron, leaves a small trace in the afferent synaptic weights of that neuron. As a result, the same neuron is more likely to fire again when a similar input pattern occurs in the future, which leads to further strengthening of the synaptic weights. Due to the combined effect of synaptic plasticity and local competition among neurons, network neurons start specializing on different salient input patterns of their respective 6 × 6 input fields (Fig 3F). Homeostatic intrinsic plasticity ensures during this learning process that neurons which specialize on weak patterns (weak synaptic weights) are not disadvantaged compared to neurons which focus on strong patterns (strong synaptic weights), by regulating intrinsic excitabilities such that all network neurons maintain their long-term average activity *m*_*k*_. An example that illustrates how homeostatic intrinsic plasticity contributes to synaptic learning of input patterns with different intensities is provided in *Illustration of learning with homeostatic intrinsic plasticity* in Methods.

A direct consequence of the gradual specialization of network neurons on local salient input patterns is that the network response becomes increasingly more structured and reliable during learning (Fig 3G, top): each network neuron becomes a *probabilistic local expert* for one of the salient activity patterns in its local 6×6 input field. This assigns a particular meaning to each of the random variables *z*_*k*_ of the network: The activity of neuron *z*_*k*_ represents the presence or absence of the salient local input feature which is encoded in its afferent weights *V*_*ki*_. Nearby network neurons (such as the blue/red/green neurons in Fig 3A) are in competition due to lateral inhibition. Therefore, whenever an input is presented to the network, the activity in the local 6×6 input field is effectively categorized by similarity to the preferred patterns of the three neurons. This is visualized in Fig 3E: Each dot in the 2D-projection represents one instantiation of the local 6×6 input field highlighted in Fig 3A. Grey dots indicate that none of the three neurons (green/red/blue) fired in response to the local input (≈ 80% of the time in accordance with the targets *m*_*k*_). Colored dots indicate that the respective network neuron fired (colors as in Fig 3A). After learning, the input space is segmented into three regions (Fig 3E, right). Ambiguous input instances at borders between regions evoke probabilistic responses (e.g. between blue and red region). In this case, the network stochastically responds with one of the two (or three) possible interpretations in order to approximate the posterior probabilities of each hidden cause. The resulting representation on the network level is a sparse structured spike response that conveys highly compressed information about the input.

The qualitative changes in network behavior described above are paralleled by a quantitative improvement of network performance measured by the log-likelihood ℒ(***θ***) (Fig 3C), as predicted by Corollary 2: From the perspective of statistical model optimization, the learning dynamics due to synaptic and homeostatic plasticity guide a local stochastic search in parameter space which on average increases the lower bound ℱ of the log-likelihood ℒ(***θ***). At the end of learning, after *T* = [10, 000] *s*, the network has identified a faithful representation of the actually presented local activity patterns (see Fig 3D). For the shown comparison, theoretically optimal weights were calculated from the presented activity patterns according to Eq (9). The reason for the small but systematic differences (learned weights are a bit stronger than predicted) can be found in the facts that the network samples from a variational posterior distribution and that the plasticity rules (12) and (13) optimize a low bound ℱ instead of the log-likelihood ℒ.

In summary, we have demonstrated in this subsection how a neural-sampling network can adapt its internal parameters to perform probabilistic inference on distributed spiking input streams. The theoretically derived plasticity rules (12) and (13) enable the sampling network to develop a sparse and reliable spike code that carries the most salient information of the input stream. This statistical optimization process evolves in a fully self-organized manner by turning network neurons into probabilistic local experts that compete in explaining the presented spike input according to the rules of probability theory.

#### The distinct role of lateral inhibition for synaptic learning

Before we address plasticity of recurrent synapses, an important contribution of inhibition to synaptic learning deserves a brief discussion. The simplicity of the synaptic plasticity rule (12) arises from the salient lateral inhibition network motif. To identify the role of lateral inhibition for synaptic learning, it is instructive to review the derivation of (12) in the absence of inhibition. By repeating the derivation with ***W*** ^inh^ = **0**, we obtain:

$$\frac{\partial }{\partial t}\;V_{ki}=\eta_{V}\cdot z_{k}\left(t\right)\cdot [y_{i}\left(t\right)-\sigma \;(V_{0i}+\sum_{j=1}^{K}V_{ji}\;z_{j}\left(t\right))].$$ (15)

The sum on the right-hand side in (15) depends on the activity of neighboring cells *z*_*j*_, *j* ≠ *k*, as well as on their afferent weights *V*_*ji*_, thereby rendering statistically optimal learning non-local. In contrast, local inhibition introduces competition among nearby neurons such that each input variable *y*_*i*_ is explained by at most one hidden cause *z*_*k*_ at a time, and, as a consequence, the complex non-local term in Eq (15) vanishes. Notably, this outcome is not an artifact of the specific probabilistic model we use, but rather is a general consequence of explaining away effects in any graphical model with converging arrows. This finding suggests that lateral inhibition among nearby neurons assists synaptic learning in a Bayesian framework of model optimization. On the other hand, when lateral inhibition extends beyond neurons with shared afferent input, the expressive power of the probabilistic model is reduced since less hidden causes *z*_*k*_ are allowed to be active simultaneously. These theoretical considerations suggest that the emergence of efficient representations benefits from network architectures in which the range of local inhibition matches the spatial extent of excitatory cells that share common afferent input.

### Plastic recurrent synapses integrate structural knowledge

We have demonstrated how synaptic plasticity can guide statistically optimal learning of afferent connections *V*_*ki*_. This learning process led to the emergence of probabilistic local experts. As a result, the configuration ***z***(*t*) of active network neurons indicates the subset of currently present local features in the spiking input ***y***(*t*). In most biologically relevant scenarios, these local input features are unlikely to be statistically independent. For early visual areas, for instance, we can expect the input in nearby spatial receptive fields to exhibit some degree of correlation. Similarly, across sensory modalities certain visual, auditory or tactile stimuli will often occur together. These statistical correlations among local features give rise to non-vanishing covariances 〈*z*_*k*_ *z*_*j*_〉 − 〈*z*_*k*_〉 〈*z*_*j*_〉 in the network. In the probabilistic model *p* (***y***, ***z*** ∣ ***θ***), the covariance between network neurons is determined by the recurrent weights $W_{kj}^{\text{exc}}$ which shape the prior distribution *p*(***z*** ∣ ***θ***). Since a probabilistic model supports inference best if its prior *p*(***z*** ∣ ***θ***) reflects the input-evoked correlation structure [38], we extend our investigation of statistically optimal learning to plastic recurrent synapses $W_{kj}^{\text{exc}}$. For the derivation, we can follow the same approach that has already afforded the plasticity rule Eq (12) for afferent synapses. The key idea is to identify a synaptic plasticity rule that points on average in the direction of $\partial ℱ/\partial W_{kj}^{\text{exc}}$, i.e., in the direction of increasing ℱ. The derivation is provided in *Model optimization via Generalized Expectation Maximization* in Methods and yields the following theoretically optimal plasticity rule for recurrent weights:

$$\frac{\partial }{\partial t}W_{kj}^{\text{exc}}=\eta_{W}\;\cdot \;\left(z_{k}\left(t\right)\;z_{j}\left(t\right)-\phi_{kj}^{\text{opt}}\;\left({\hat{W}}^{\text{exc}},\;{\hat{W}}^{\text{inh}},\;\hat{b}\right)\right)$$ (16)

with learning rate *η*_*W*_ and $\phi_{kj}^{\text{opt}}\left({\hat{W}}^{\text{exc}},{\hat{W}}^{\text{inh}},\hat{b}\right)={\langle z_{k}z_{j}\rangle }_{p\left(z\;|\;\theta \right)}$. The plasticity rule Eq (16) features a long-term potentiation (LTP) and a long-term depression (LTD) term: Concurrent activation *z*_*k*_(*t*) *z*_*j*_(*t*) of network neurons in response to the input strengthens the synapse in a local Hebbian LTP update. Depression, however, turns out to be non-local since the term $\phi_{kj}^{\text{opt}}$ depends on all parameters ${\hat{W}}^{\text{exc}}$, ${\hat{W}}^{\text{inh}}$ and $\hat{b}$ of the prior distribution *p*(***z*** ∣ ***θ***). This increased complexity of learning in recurrent systems is well-known in machine learning theory [39], and we can employ (non-local) machine learning techniques to determine the value of $\phi_{kj}^{\text{opt}}$ in a so-called sleep phase. This algorithmic approach is known as wake-sleep learning in the literature [40]. Since the calculation of $\phi_{kj}^{\text{opt}}$ is often computationally costly, the development of approximate solutions (such as contrastive divergence [41, 42]), which are tailored to particular network architectures and learning tasks, turned out beneficial in machine learning. The question, whether the brain makes use of similar learning strategies, is subject of ongoing theoretical and experimental research (see e.g. [43, 44], or [45] for a recent review), and it appears indeed conceivable that nature found ways to estimate $\phi_{kj}^{\text{opt}}$.

In the present study, we contribute to this intriguing hypothesis by exploring to what extent even simple plasticity rules could be sufficient to approximate the non-local plasticity dynamics of Eq (16) in the neural sheet model. Specifically, we are interested in plasticity rules which (i) rely on only local information, (ii) can be applied uniformly to all recurrent synapses, and (iii) are shaped by only a small set of parameters. In case of the architectures often considered in the machine learning literature (stacked Restricted Boltzmann Machines (RBMs) and variants thereof) this would likely be a hopeless endeavor. However, the network architecture of the neural sheet is fundamentally different from stacked RBM architectures, and the local integration of input may further ease the complexity of recurrent learning. The conceptual separation of feature detection (through plastic afferent weights *V*_*ki*_) on the one hand, and feature structure (through plastic recurrent weights $W_{kj}^{\text{exc}}$) on the other hand, can be expected to facilitate learning in that—once the essential features have been identified—the complexity of learning the prior shows some resemblance to the reduced complexity of training a fully visible Boltzmann machine [46]. Thus, the emergence of probabilistic local experts may provide a guidance for learning of recurrent connections. Inspired by the structure of Eq (16) and the weight dependence of the LTD term in Eq (12), we make the following ansatz for a local synaptic plasticity rule:

$$\frac{\partial }{\partial t}W_{kj}^{\text{exc}}=\eta_{W}\;\cdot \;\left(z_{k}\left(t\right)\;z_{j}\left(t\right)-\phi_{\vartheta }\left(W_{kj}^{\text{exc}}\right)\right)$$ (17)

where the LTD-term $\phi_{\vartheta }(W_{kj}^{\text{exc}})$ only depends on the weight of the respective synapse, and the parameters ***ϑ*** of *ϕ*_***ϑ***_ are to be determined for the given learning task. Ideally, the LTD function *ϕ*_***ϑ***_ should be chosen such that Eqs (16) and (17) lead to the same weight values $W_{kj}^{\text{exc}}$ under given neuronal spike patterns. In the simple rule, plasticity is governed by two antagonistic terms: LTP is proportional to the average coactivation 〈*z*_*k*_ *z*_*j*_〉 of a synapse’s pre- and post-synaptic neurons; LTD is proportional to $\phi_{\vartheta }(W_{kj}^{\text{exc}})$. Hence, if *ϕ*_***ϑ***_ is a continuous and monotonically increasing function in $W_{kj}^{\text{exc}}$, there exists an equilibrium weight $W_{kj}^{\text{exc}}$ for each value of the coactivation 〈*z*_*k*_ *z*_*j*_〉. Based on this observation, a principled approach to identify a suitable LTD function *ϕ*_***ϑ***_ is to first determine optimal weights $W_{kj}^{\text{opt}}$ for a given learning problem using the theoretically optimal wake-sleep rule (16), and then to fit $\phi_{\vartheta }(W_{kj}^{\text{exc}})$ such that the identified optimal weights are approximately reproduced under given coactivations 〈*z*_*k*_ *z*_*j*_〉 by the simple rule (17). In *Approximate plasticity rule for recurrent synapses* in Methods, we show how for a set of optimal weights $W_{kj}^{\text{opt}}$ and corresponding coactivations 〈*z*_*k*_ *z*_*j*_〉 an LTD function $\phi_{\vartheta }(W_{kj}^{\text{exc}})$ can be constructed that features the desired convergence points $W_{kj}^{\text{opt}}$. It must be noted that the local plasticity rule (17) can only serve as a heuristic for approximating the plasticity dynamics of the theory-based wake-sleep rule (16). As such, the parameters ***ϑ*** need to be adjusted for every learning scenario and there exists no strict guarantee of stable plasticity dynamics. Therefore, we explored the suitability of the local recurrent plasticity rule in computer simulations by comparing the learning results of the local rule with the outcome of wake-sleep learning.

#### Demonstration of self-organized integration of structural knowledge

**Fig 4:**
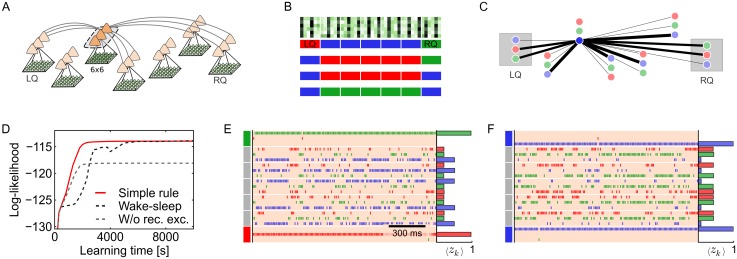
Plastic recurrent synapses integrate structural knowledge. (A) Network architecture with seven recurrently connected local populations (all-to-all beyond inhibition). Input weights *V*_*ki*_ and recurrent excitatory weights $W_{kj}^{\text{exc}}$ are plastic. One of three stripe activity patterns (visualized as red/green/blue) is presented as spiking input to each local population. (B) Examples of the input structure. Top: input activity of the strip patterns (250 ms average). Bottom: The two outer locations serve as left and right cue (LQ,RQ). Cue input patterns are chosen independently. The cues determine the type (“color”) of the inner patterns in that the inner inputs are always different from both cues. In addition, inner patterns are always consistent. Hence, inner network neurons must consider both cues and the state of the other inner network neurons to infer their own state. (C) Recurrent weights of the highlighted blue-tuned neuron after 25, 000 s of learning with the simple local plasticity rule (17). Network neurons are colored according to the local pattern they have become experts for. Line width encodes the synaptic weight $W_{kj}^{\text{exc}}$ (min. line width 0.2 for $W_{kj}^{\text{exc}}=0$). In accordance with the input structure, the inner blue neuron has developed strong excitatory connections to the other inner blue neurons and moderately strong connections to the red and green cue neurons. (D) Comparison of the log-likelihood over the first 10, 000 s of learning for three recurrent plasticity conditions: with the simple recurrent plasticity rule (red), with the wake-sleep algorithm (dashed black), and without recurrent excitation (dashed gray). Recurrent plasticity significantly enhances the network’s learning capabilities. (E) The knowledge on the input structure, that was learned by the recurrent synapses $W_{kj}^{\text{exc}}$, enables inference of correct global network states in face of incomplete input. Only the outer cues are presented while all inner inputs show an uninformative (“gray”) pattern. With red and green cues, the network correctly infers that the inner hidden causes should be blue, most of the time. Horizontal bars: mean activity 〈***z***_*k*_〉 of network neurons (average over 100 s). (F) When both outer cues are blue, the already incomplete input is furthermore ambiguous. During inference, the network switches stochastically between the two consistent global interpretations.

We tested the learning capabilities of the simple plasticity rule (17) and compared it with the theoretically optimal wake-sleep rule (16) in a computer simulation with seven spatially separate network populations (see Fig 4A). Each population consisted of three neurons and received spiking input from a group of 6×6 inputs. Within each population neurons are subject to lateral inhibition. Neurons from different populations are linked via all-to-all recurrent excitatory connections. At each spatially separate 6×6 input location one of three local activity patterns may occur. The local activity patterns are simple stripe patterns (see Fig 4B top) that can easily be learned by the afferent weights *V*_*ki*_. For brevity, we refer to the three activity patterns as the ‘red’, ‘green’ and ‘blue’ pattern in the following.

In order to assess the network’s ability to detect and integrate highly interdependent correlation structures among features at different locations, we introduced complex dependencies between the input presented to the seven populations: While the two *outer* input locations served as a left cue (LQ) and right cue (RQ), and were chosen independently, the five *inner* locations were chosen to be (a) different from the cues, and (b) consistent with each other. For instance, if the outer cue patterns were red and green, all inner input patterns were blue. If, however, both cues showed the same pattern (e.g., both blue), an ambiguous situation arose: In this case, the inner patterns were still chosen to be different from the cue, but additionally all inner patterns were consistently of the same type (either all red or all green). Several examples illustrating this correlation structure are sketched in Fig 4B. As a consequence, an inner network neuron, that is tuned to one of the three local patterns, has to consider both cues and the state of all other inner network neurons in order to infer its own state correctly.

We tested the network’s ability to recover the statistical structure of the input in a simulation with concurrent learning of afferent weights and recurrent weights (initial values: $V_{ki}=W_{kj}^{\text{exc}}=0$ and restricted to positive values). In a first simulation run, recurrent synaptic plasticity followed the theoretically optimal wake-sleep plasticity rule (16). As expected from the theory, all network neurons developed a tuning to one of the local input patterns, and recurrent weights correctly reflected the correlation structure of the task after 25, 000 s of learning: Similarly tuned inner network neurons formed strong excitatory recurrent links $W_{kj}^{\text{exc}}$; in addition, inner neurons developed excitatory connections with compatible cue neurons (e.g. with the red and green cue for a blue-tuned inner neuron). The ongoing adaptation of the network is reflected in the log-likelihood function ℒ(***θ***) shown in Fig 4D. The black dashed line shows the learning progress with the theoretically optimal learning rule. For comparison, we performed an independent simulation with recurrent plasticity switched off (dashed gray, all $W_{kj}^{\text{exc}}$ fixed at zero). Without recurrent excitation, the log-likelihood settled at a significantly lower value. The gap in ℒ between the two simulations corresponds to the structural information stored in the prior *p*(***z*** ∣ ***θ***).

Building on the theory-based learning result, we constructed a suitable LTD function *ϕ*_***ϑ***_ to obtain a simple plasticity rule. The set of target weights $W_{kj}^{\text{opt}}$, that emerged during the first simulation run, and the set of coactivations 〈*z*_*k*_ *z*_*j*_〉, that had led to these weights, are provided in *Details to the computer simulations* in Methods. Based on the observed functional dependence between $W_{kj}^{\text{opt}}$ and 〈*z*_*k*_ *z*_*j*_〉, we chose the following LTD function:

$$\phi_{\vartheta }\left(W_{kj}^{\text{exc}}\right)=m_{k}\;\cdot \;m_{j}+\frac{1}{\gamma }\text{tan}\;\left(\frac{\pi }{2}\frac{W_{kj}^{\text{exc}}}{W^{\text{max}}}\right)$$ (18)

with parameters ***ϑ*** = (*W* ^max^ = 1.41, *γ* = 31.6) fitted to the data. The LTD function (18) has two components: The term *m*_*k*_ ⋅ *m*_*j*_ describes the expected coactivation 〈*z*_*k*_ *z*_*j*_〉 if the neurons were statistically independent. This case is associated with weight $W_{kj}^{\text{exc}}=0$ when synaptic plasticity follows Eq (17). The second term accounts for positive correlations between *z*_*k*_ and *z*_*j*_ through a stabilizing weight dependence. While the specific tangent-shape is a heuristic, the functional form has some properties that are generally expected for Hebbian-type plasticity: The LTD function is strictly monotonically increasing such that higher coactivations settle at stronger efficacies; and, the maximum efficacy of a synapse is bounded since the LTD contribution goes to infinity as $W_{kj}^{\text{exc}}$ approaches *W* ^max^. The scaling parameter *γ* sets the strength of LTD.

In a second simulation run, all recurrent and afferent weights were reset to zero, and recurrent synaptic plasticity followed the simple local plasticity rule given by Eqs (17) and (18). As seen in Fig 4D, the simple rule is virtually indistinguishable from the theoretically optimal rule in terms of the log-likelihood. The resulting recurrent connectivity structure after 25, 000 s of learning is shown in Fig 4C for the example of an inner network neuron that has specialized on the blue input pattern: The neuron developed strong recurrent weights with all other inner neurons of similar tuning, and moderately strong weights with red- and green-tuned cue neurons. This connectivity matrix mirrors the connectivity structure obtained with theoretically optimal wake-sleep learning (see *Details to the computer simulations* in Methods for numerical values).

The learned structural knowledge can be exploited by the sampling network during inference. Most distinctly, the benefits of well-adapted recurrent excitatory connections become apparent in response to incomplete, or even ambiguous, stimuli (Fig 4E and 4F). To emulate a scenario of incomplete observations, only outer cues were presented to the network while all inner inputs showed uninformative, uniform activity (indicated as gray). For example, when the cues are set to red and green, as shown in Fig 4E, the network correctly infers that all inner features should be blue most of the time. In the given experimental protocol, this knowledge can only be communicated via recurrent excitatory synapses. In a second example shown in Fig 4F, the already incomplete input is furthermore chosen to be ambiguous by presenting a both-blue cue. Hence, the inner features should be either all red or all green. In this case, the network activity stochastically switches between the two inferred consistent interpretations (note that the switching of network neurons at inner locations is synchronized), as expected for sampling from a bi-modal posterior distribution *p* (***z*** ∣ ***y***, ***θ***).

In conclusion, the computer experiment in Fig 4 demonstrates that the sampling network model can concurrently identify salient features of its input stream and recover complex correlations among spatially distributed features through synaptic plasticity. Self-organized learning of recurrent excitatory connections can be understood as an ongoing refinement of the prior distribution in the network’s internal model of presented input. The obtained structural knowledge on the input distribution significantly enhances the network’s ability to maintain globally coherent network states in face of incomplete and ambiguous observations. For the examined inference task, which required integration of two independent cues as well as the state of multiple other network neurons, we observed that even the simple local plasticity rule (17) + (18) endowed the network with close-to-perfect learning capabilities. This observation indicates that the architecture of the neural sheet model eases the complexity of recurrent learning in that the local input integration through probabilistic experts can guide learning of recurrent connections. However, as a word of caution, owing to theoretical considerations it is unlikely that such simple plasticity rules will be sufficient to solve arbitrarily sophisticated learning problems. For instance, probabilistic causal relations of the type “A often implies B” but “B does not cause A” lie beyond the expressive power of the employed single-layer associative prior (6). Yet, as shown in [47], it is possible to implement such probabilistic relations into spiking neural networks by using complex connectivity structures and asymmetric recurrent weights. Regarding the ability of the local plasticity rule (17) to approximate the theoretically optimal rule (16), it can be expected that reliable learning is limited to scenarios where only few recurrently connected neurons are typically active simultaneously. For instance, it is unlikely that deep learning architectures with additional hidden units (without synaptic input connections) could be trained with the simple plasticity rule.

### Emergence of excitatory subnetworks in neural sheets

In previous subsections, we have examined how small sheets of spiking neurons with local lateral inhibition and recurrent excitation can perform probabilistic inference by sampling from the posterior distribution *p* (***z*** ∣ ***y***, ***θ***) of a well-defined probabilistic model, and how the parameters ***θ*** of the underlying probabilistic model can be adapted to the presented input by plastic afferent and recurrent synapses. For illustration purposes, network architectures were small and computer simulations were tailored to highlight specific aspects of sample-based inference and statistical learning.

**Fig 5:**
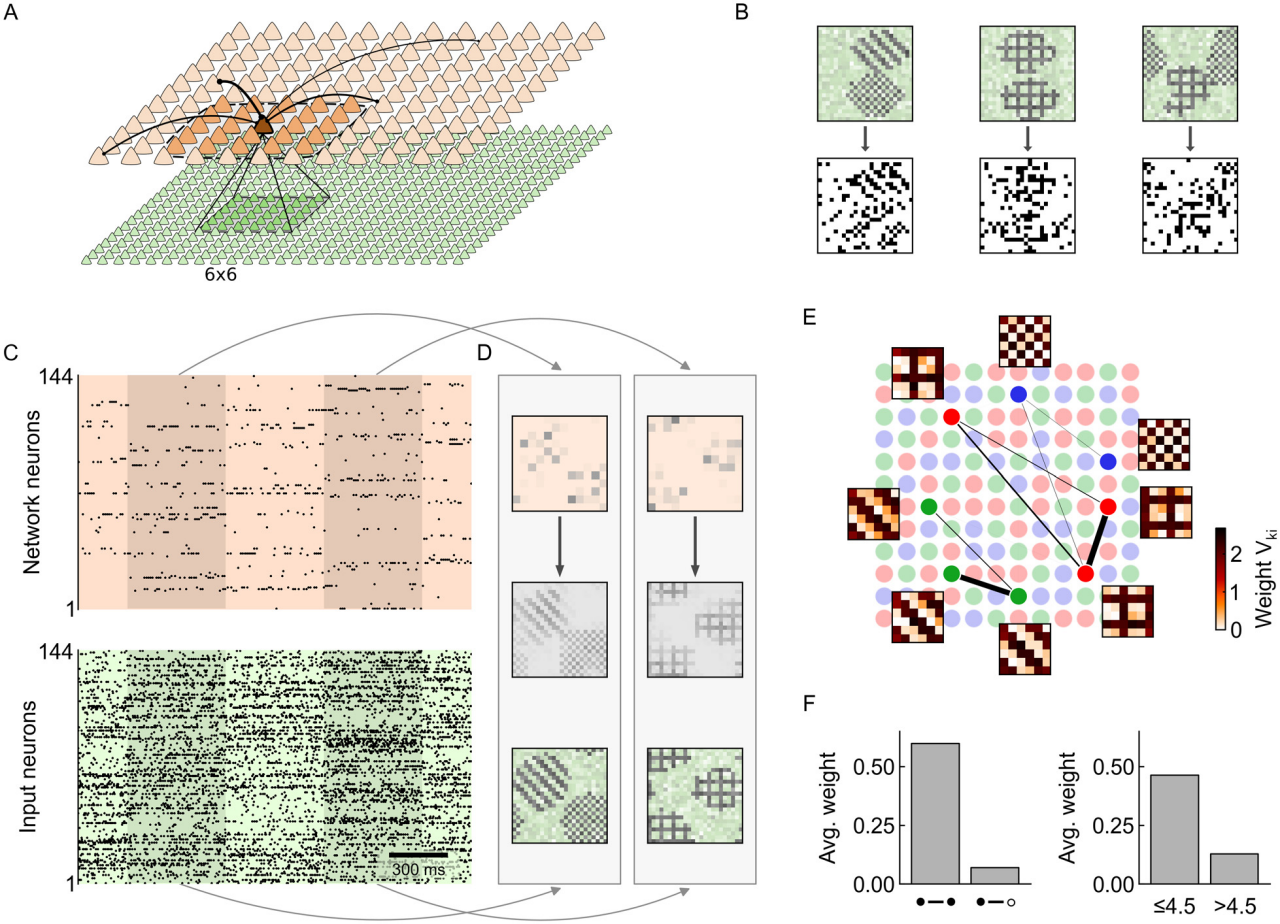
Emergence of excitatory subnetworks in neural sheets. (A) Network architecture with 24×24 inputs and 12×12 network neurons. Each network neuron receives input from a subset of 6×6 inputs, as illustrated for the highlighted neuron. Input subsets of neighboring neurons are shifted by two. The dashed line marks the range of lateral inhibition for the highlighted neuron. Additionally, pairs of network neurons, that do not inhibit each other, maintain sparse recurrent excitatory synapses $W_{kj}^{\text{exc}}$ (25% prob. for a reciprocal connection). All bottom-up weights *V*_*ki*_ and recurrent weights $W_{kj}^{\text{exc}}$ are plastic. (B) Three exemplary input activity patterns (top; 500 ms average) and spiking input instantiations ***y***(*t*) therefrom (bottom). Two of three local input templates (grid/diagonals/checker) are presented at different random locations at a time. (C) Input spike trains (bottom) and network response (top) after 10, 000 s of learning. Input patterns switch every 500 ms. Only a random subset of 144 inputs is shown to facilitate the comparison of spike density. The network response is considerably sparser than the input. (D) Reconstruction of the input from the sparse network response. Bottom: Input 〈***y***(*t*)〉 averaged over the highlighted periods in C. Top: Average network response 〈***z***(*t*)〉 during the same periods. Middle: Reconstruction 〈***y*** ^gen^〉 of the input from the network response by means of the learned generative model. (E) Emergence of distributed assemblies of local experts. During learning each network neuron becomes a probabilistic expert for one of the local activity patterns (indicated by red/green/blue color). Input weights *V*_*ki*_ are shown for highlighted neurons. In addition, the network developed recurrent excitatory connections $W_{kj}^{\text{exc}}$. Neurons, that are experts for similar patterns, form excitatory subnetworks after learning (line width proportional to $W_{kj}^{\text{exc}}$). (F) Recurrent excitatory connections between similarly tuned neurons are on average considerably stronger than connections between neurons with different specialization (left). In addition, connections between nearby pairs of network neurons are stronger than between distant pairs (Euclidean distance; based on 4198 recurrent exc. synapses).

In this final subsection, we combine all mechanisms described above and explore Bayesian information processing and self-organized learning in a spatially extended neural sheet model that is exposed to multiple local input streams in parallel. The employed network architecture is shown in Fig 5A. Network neurons are organized in a two-dimensional lamina and integrate spiking input locally via plastic afferent synapses (initial weight *V*_*ki*_ = 0). Lateral inhibition is spatially confined for any point of reference (dashed line for the highlighted cell) yet omnipresent in the homogeneous continuous tissue, giving rise to a plethora of interleaved and overlapping competitive subcircuits. Beyond the range of inhibition, network neurons maintain sparse, plastic excitatory connections (per pair: 25% chance of a reciprocal connection, initial weight $W_{kj}^{\text{exc}}=0$). Note that, due to the sheer amount of possible network states ***z***(*t*), a traditional analytical calculation of the posterior distribution *p* (***z*** ∣ ***y***, ***θ***) becomes intractable in this architecture.

We presented spiking input to the network as shown in Fig 5B: Three local activity motifs (grid, checkerboard, diagonal stripes; top row of Fig 5B) can appear at different locations in the input space. Each local motif is larger than the diameter of afferent connections *V*_*ki*_ to individual network neurons (highlighted green neurons in Fig 5A). At any time, two randomly drawn motifs are presented at different non-overlapping random locations. Inputs *y*_*i*_ that are not part of a local motif maintain a low-activity background firing rate. Poisson spike trains were generated from the resulting rate patterns (top row of Fig 5B), leading to versatile (and seemingly noisy) input ***y***(*t*) that was presented to the network (bottom row of Fig 5B).

This input mimics some important aspects of cortical information processing: First, input neurons are tuned to certain input features (the presence/absence of the three input motifs), but their spiking activity is highly stochastic and the same input neuron *y*_*i*_ can be responsive to multiple different input patterns. Consequently, inputs *y*_*i*_ are not very informative when observed individually. Second, input motifs are too large to be explained by a single network neuron, and thus multiple network neurons must be recruited in parallel for the explanation. Finally, multiple independent streams of information can be presented to the network at different locations simultaneously.

We tested the capability of the neural sheet to integrate and adapt to this input in a 10, 000 s learning experiment. All parameters $\left(W_{kj}^{\text{exc}},V_{ki},b_{k}\right)$ were plastic. As before, we first trained the network with the theoretically optimal recurrent plasticity rule (16). Fitting the parameters ***ϑ*** for the simple rule (17) + (18) yielded *W* ^max^ = 2.70 and *γ* = 734. Note that, even though the two parameters ***ϑ*** are tailored to the learning task and are thus assumed to be given to the network, the resulting plasticity rule *ϕ*_***ϑ***_ is of drastically reduced complexity compared with wake-sleep learning. In particular, all recurrent synapses share the same LTD function $\phi_{\vartheta }(W_{kj}^{\text{exc}})$. Using the fully-local plasticity rules for $W_{kj}^{\text{exc}}$, *V*_*ki*_, and *b*_*k*_, the network was then simulated again “from scratch”. Fig 5C shows spike trains of the input and the network neurons after learning. To facilitate the comparison of the network response to the input, only a random subset of 144 input neurons is shown. The network response is considerably sparser than the input, with only few network neurons firing simultaneously at a long-term average firing rate of ca. 2.5 Hz. To examine what aspects of the input are conveyed by the sparse response, we can adopt the generative perspective, again. From the learned afferent weights *V*_*ki*_, the translation to the input domain as established by Eq (9) and the likelihood model *p* (***y*** ∣ ***z***, ***θ***), we can reconstruct the average input 〈***y*** ^gen^〉 expected from the network response ***z***(*t*) during a short time window:

$$\langle y_{i}^{\text{gen}}\rangle =\frac{1}{\Delta }\int_{t_{0}}^{t_{0}+\Delta }\sigma \;\left(V_{0i}+\underset{k}{\sum }z_{k}\left(t\right)V_{ki}\right)\;dt,$$ (19)

where we chose Δ = 500 ms. This reconstruction is shown in Fig 5D for the two highlighted time windows of Fig 5C and as a video (S1 video) for a longer time span with Δ = 100 ms. The reconstructed 〈***y*** ^gen^〉 are plotted in gray scale alongside the true average inputs 〈***y***(*t*)〉 plotted in green. While small differences, especially at the spatial feature boundaries, are visible, the network response still conveys the most salient information of the presented input. In other words, the network has developed a code which is not only sparse but also very efficient, carrying highly compressed information about the presented input spike patterns in only few spikes.

Fig 5E provides insight into how this code has been established: Each network neuron has become a local expert for one of the three local inputs motifs, and together, network neurons cover the entire input and feature space. The afferent weight values *V*_*ki*_ are shown as small colorbar insets for the 6 × 6 input fields of the highlighted network neurons in Fig 5E. Excitatory connections among network neurons store positive associations, i.e., a strong excitatory weight between two network neurons indicates that the corresponding local features tend to co-occur in the input. This co-occurrence mirrors the local nature of the presented input motifs: The presented motifs are larger than the 6 × 6 input field of a network neuron. Consequently, multiple nearby network neurons turn active simultaneously in the posterior for explaining a presented pattern. The systematic co-activation of network neurons triggers LTP in their recurrent connections. In line with this notion, we observe the emergence of recurrent excitatory subnetworks among those neurons that receive input from contiguous spatial domains and exhibit tuning to similar input features. These learned associations can be used during inference for ensuring that more likely configurations of network states are preferentially visited by the network in case of uncertainty. The qualitative observation of excitatory subnetworks is further supported by the statistical analysis shown in Fig 5F: Synaptic weights between network neurons with similar tuning are on average stronger than weights between neurons with different tuning properties (left). And, synapses between nearby cells are stronger than connections between distant cells (right), reflecting the local nature of the presented input.

Here, we have demonstrated sample-based inference and self-organized learning in a neural sheet model with 12 × 12 network neurons. The rather limited network size in Fig 5 is owing to the extensive computing time required for simulating long-term learning experiments. Notably, the theory of the neural sheet model supports networks of arbitrary size. When increasing the network size, two types of scaling can be distinguished: (a) increasing the *spatial area* covered by the neural sheet, and (b) increasing the local *density of neurons* within the range of lateral inhibition. Scaling the spatial area is expected to not significantly change any of the network dynamics during inference and learning in most scenarios. In contrast, increasing the neuron density will lead to a very sparse spike response since lateral inhibition permits only one local network neuron to be active at a time. While a very sparse response is fully covered by the theory, it could become a concern in neurobiological modeling. We will come back to this point in the Discussion section.

## Discussion

We have proposed a spatially structured spiking network model for distributed Bayesian inference and self-organized learning through synaptic plasticity. Building on the theory of MCMC sampling, we have shown how the transient spike response of the recurrent neural network can be understood as an ongoing sampling process from a well-defined Bayesian posterior distribution. Our study extends work by Buesing et al. in that it endows the generic network architecture of [17] with a spatial structure (namely, local lateral inhibition and sparse recurrent excitation) and the ability to integrate distributed spiking input. It turned out that the local integration of distributed spiking input streams assigns a particular meaning to the previously abstract random variables of the neural circuit: Network neurons encode the presence or absence of salient input features which are stored in the network’s afferent synapses. This leads to the conception of network neurons as probabilistic local experts which are organized in a tissue of interwoven local winner-take-all circuit motifs. Beyond the range of lateral inhibition, network neurons communicate via sparse recurrent excitatory connections on an intermediate spatial scale. From a theoretical perspective, the recurrent transfer of information is linked to the prior distribution in Bayes rule and captures structural knowledge on statistical correlations among spatially separate input features. This ability to align instantaneous observations with previously obtained structural knowledge according to the rules of probability theory enables the neural sheet to maintain coherent global network states even when the presented input is incomplete or ambiguous. Moreover, having a full probabilistic description *p*(***y***, ***z*** ∣ ***θ***), that covers both the input and the network response, at hand permitted a rigorous mathematical treatment of self-organized learning. Extending work of [36] and [25], we demonstrated that the interplay of STDP-type synaptic and homeostatic intrinsic plasticity can approximate stochastic online Expectation Maximization, a powerful machine learning algorithm. This is a remarkable finding: Global statistical model optimization can be achieved in a spatially extended network through only local information exchange (pre- and post-synaptic spiking activity) in a fully unsupervised manner.

### Theoretical aspects of information processing in the neural sheet

The theoretical analysis of inference and learning in the neural sheet model revealed a distinct functional role of individual network components. These are briefly addressed in the following and comprise the functional contribution of precise relative spike timing, the lateral inhibition connectivity motif, and the dynamics of self-organized synaptic learning.

#### The relative spike timing carries essential information

In the proposed neural sheet model, multivariate posterior distributions *p* (***z*** ∣ ***y***, ***θ***) are represented by interpreting network spikes as realizations of random variables *z*_*k*_ according to Eq (1): After a spike of the k-th network neuron, the associated RV *z*_*k*_ turns active for a short time *τ* (10 ms in this study). This gives rise to vector-valued network states ***z***(*t*) in which multiple RVs *z*_*k*_ will typically be active, simultaneously. As a direct consequence, the relative spike timing of neuronal subgroups carries important information on a millisecond time scale: overlapping on-times encode the probability of coactivation of the associated RVs in the multivariate distribution *p* (***z*** ∣ ***y***, ***θ***). The computational importance of incorporating coactivations during inference becomes particularly evident in ambiguous situations that support multiple coherent—but mutually exclusive—explanations. The sampling network’s spike response in such an ambiguous scenario was demonstrated in Fig 4 where the network switched stochastically between two coherent perceptual modes in a synchronized manner. The information density conveyed by this structured, bimodal response goes far beyond the quality of conclusions that could be drawn from just observing the average firing of individual neurons. Figuratively, considering only marginal responses would disregard the insight that an obstacle could be circumvented either on the left hand or the right hand side, but would instead suggest to steer a happy medium.

#### Lateral inhibition facilitates local synaptic learning

This information-rich neural representation of globally coherent network states was shown to emerge fully autonomously through the interplay of local synaptic and intrinsic plasticity rules. The derivation of the weight-dependent plasticity rule (12) for afferent synapses *V*_*ki*_ furthermore revealed an essential role of the local lateral inhibition network motif during learning. The contribution of lateral inhibition becomes apparent when the derivation of Eq (12) is repeated in the absence of the inhibition motif, resulting in the update rule (15). The latter rule (15) requires information not only on the pre- and post-synaptic spiking activity of a plastic synapse, but also on the specific weight values of other nearby synapses, thereby rendering learning non-local. This increased complexity of parameter learning is a general problem in graphical models with converging arrows, known as explaining away, and thus, the locality of synaptic learning displays a conceptual challenge for a wide range of Bayesian network architectures. In the neural sheet model, lateral inhibition establishes a particularly strong form of explaining away by ensuring that each input *y*_*i*_ is explained by at most one network variable *z*_*k*_, at a time. The resulting competition among network neurons restores the locality of information required for synaptic learning. We therefore suspect that the competition introduced by local lateral inhibition could assist synaptic learning in a wide range of network models in that it facilitates global statistical model optimization through local synaptic plasticity rules. In particular, in order to maximize the expressive power of the system while preserving local synaptic learning, our theory suggests that self-organized learning of efficient representations benefits from network architectures in which the range of lateral inhibition matches the spatial extent of network neurons that share common afferent input.

#### Recurrent plasticity integrates structural knowledge

Regarding plasticity of recurrent connections $W_{kj}^{\text{exc}}$ in the neural sheet, we found optimal learning rules to be fundamentally non-local; a hardly surprising finding for recurrent systems. We therefore explored in computer simulations to what extent even simple local plasticity rules with approximately matching convergence properties could be sufficient to recover complex structural correlations in the presented input. In the small computer experiments of Figs 4 and 5, we observed no significant impairment in the emergent weight configuration when simple local plasticity rules were used (compared with the theoretically optimal non-local rule). This observation indicates that, for tasks of “not too high” complexity, the prior *p*(***z*** ∣ ***θ***) could indeed be adjusted by only local synaptic plasticity. The specific shape of the employed plasticity rule, however, needs to be tailored to statistical properties of the given task. For biological systems, it is thus conceivable that evolution forged tailored simple plasticity dynamics for certain neuronal populations and brain areas that interact with more elaborate plasticity types.

#### Robust evasion of suboptimal solutions

Finally, the learning dynamics of the underlying stochastic online Expectation Maximization algorithm, that performs local gradient ascent in the synaptic weights, deserves a brief discussion. Like every local optimization algorithm, learning in the neural sheet is only guaranteed to converge to a local optimum of the objective function ℱ. This is a general issue in unsupervised learning since the likelihood functions of complex probabilistic models may possess many local optima. However, two properties of the neural sheet model are expected to mitigate this issue: First, the network employs stochasticity in two ways, via the random presentation of input samples ***y*** ∼ p*(***y***) and via the stochastic nature of the network response ***z*** ∼ *p* (***z*** ∣ ***y***, ***θ***). This induces stochastic fluctuations in the synaptic weights and facilitates the evasion of local optima (compared with a fully deterministic batch algorithm as often used in machine learning). Second, homeostatic intrinsic plasticity forces all neurons to participate in explaining the input. Thereby, many “particularly bad” local optima, which recruit only a small fraction of hidden causes ***z***_*k*_ in the average posterior, are automatically evaded. In accordance with these properties, we observed in the computer simulations that learning was generally very robust and that the network reliably identified near-optimal parameter settings most of the time.

### Idealized modeling assumptions and conceptual limitations

Connectivity structure and neural dynamics of the proposed sheet model have their origin in top-down principles of Bayesian information processing and machine learning theory. In the spiking network implementation of the abstract algorithms, the time scales involved during inference and learning span multiple orders of magnitude, ranging from milliseconds for the instantaneous sampling process, over tens of seconds for homeostatic plasticity, up to minutes and hours for synaptic plasticity (see also *Interaction of time scales* in Methods). In order to keep these complex interactions in the recurrent system theoretically tractable, several idealized modeling assumptions had to be made.

We employed a simple spike response neuron model that elicits action potentials stochastically based on an idealized membrane potential. The membrane potential integrates synaptic input linearly, and synaptic transmission is mediated via rectangular non-additive post-synaptic potentials without delay. There exist many potential sources in the brain to generate stochastic spike responses, ranging from channel noise and synaptic noise to recurrent network phenomena [48]. While in-vitro data [33] justify the employed neuron model as a first approximation of stochastic neuronal responses, the interactions of noise sources on a network level are not yet sufficiently understood. Studies from computational neuroscience suggest that spiking input from external neuronal populations [19] or the activity that arises in recurrent networks with probabilistic synapses [49] could lead to the employed stochastic neuronal activation.

Homeostasis entered the system only in a single process, namely as homeostatic intrinsic plasticity, with the aim to maintain a predefined average firing activity. In order to stay close to the derived learning rules, synaptic plasticity was implemented on the level of neuronal states, i.e., on the value of *y*_*i*_(*t*) and *z*_*k*_(*t*), and was not further mapped to the level of spikes. However, in [25] and [28] it has been demonstrated that (variants of) the plasticity rule (12) can be mapped down to the spike level, and that the resulting plasticity dynamics are tightly linked to spike-timing dependent plasticity. In the computer simulations, lateral inhibition was transmitted via strong reciprocal synapses between directly connected network neurons, thereby integrating out the dynamics of putative interneurons. Regarding the connectivity structure, lateral inhibition strictly obeyed the condition that a reciprocal inhibitory connection exists between two network neurons if the neurons share common inputs. We did not investigate synaptic plasticity of inhibitory connections. In particular, the range of lateral inhibition and the size of receptive fields were treated as constant. In line with the generic probabilistic model of [17], that was employed for the network population, recurrent excitatory connections were symmetric. On a more conceptual level, it is noteworthy that, while the sampling process of the network evolves in time, the underlying probabilistic model *p*(***y***, ***z*** ∣ ***θ***) is inherently non-temporal, i.e., the theory makes no predictions on the temporal structure of network trajectories or the integration of salient temporal features of the input. Possible extensions of the theory in order to support more complex recurrent (and also asymmetric) connectivity structures, soft lateral inhibition, as well as the integration of temporal sequences are outlined below.

### Relation to cortical microcircuits

The proposed neural sheet model shares some striking similarities with cortical microcircuits. These similarities range from salient connectivity motifs, to microscopic neural dynamics of single cells and synapses, up to population-level response characteristics in living animals. Most clearly, the neural sheet model can be linked to salient aspects of cortical layer 2/3, with network neurons being associated with pyramidal cells and lateral inhibition being mediated disynaptically by fast-spiking interneurons (e.g., basket cells). Certainly, the idealized neural dynamics of the model cannot be expected to find any precise counterpart in biology. And, of course, the abstract network model does not (and could not) intend to provide any complete description of all the subtleties found in cortical microcircuits. Yet, we believe that the evident similarities, as briefly reviewed in the following, could contribute to sharpening our conception of the complex neural dynamics observed in living tissue.

#### Disynaptic inhibition establishes local competition among pyramidal cells

In vitro experiments indicate that central aspects of the ubiquitous lateral inhibition network motif in the neural sheet model are established in layer 2/3 by soma-targeting, fast-spiking (FS) interneurons. FS interneurons preferably form synapses locally [31] and show particularly high connection probability with nearby pyramidal cells (PCs) [50]. These GABAergic connections typically involve many (ca. 15) contacts [34] per cell-pair and inhibit the target close to its cell body [34]. In addition, FS-PC connections were reported to feature very short transmission delays of only approx. 1ms [50]. The dense and fast inhibition of PCs by FS interneurons is complemented with an increased probability for reciprocal connections [51], i.e., bidirectional links between nearby PC-FS pairs. Notably, reciprocal PC-FS connections were reported to be especially strong in either direction (see [51, 52] for PC → FS, and [51] for PC ← FS). Furthermore, also the PC → FS connection was found to have below-average transmission delays [50]. This led the authors of [50] to the conclusion that, *“[t]aken together, our in vitro data (this study) and our related in vivo data* [52] *suggest that disynaptic inhibition driven by FS GABAergic neurons in the neocortex mediates competition among excitatory neurons such that perhaps only a small fraction of excitatory layer 2/3 neurons can be active at any given time.”* The finding of lateral competition among PCs in layer 2/3 was also confirmed in vivo by [53].

#### Pyramidal cells are clearly tuned while local lateral inhibition is unspecific

In vivo studies with awake animals characterized typical response properties of PCs and interneurons. During quiet wakefulness, PCs show a much lower firing activity than FS cells [54]. This was explained by the stronger synaptic drive required for exciting PCs compared with FS cells, which maintain an average membrane potential close below threshold [54]. Furthermore, detailed patch-clamp recordings, that separated individual conductance contributions, pointed to significantly sharper spatial tuning of excitation than inhibition [55]. This finding is supported by studies with anesthetized mice that reported clear orientation selectivity of excitatory neurons, while GABAergic cells showed only little tuning [56, 57], but see [58]. Indeed, experimental data suggests that the stimulus dependence of interneuron responses could be explained by the integrated activity of surrounding neurons [59].

#### Sparse coding and structured connectivity of excitatory neurons

Experimental data on the spike response of PCs in layer 2/3 appears to be compatible with a sparse coding scheme. For instance, it was observed in vivo by [60] that even neurons with overlapping receptive fields showed only low correlation. Also [61] reported generally weak noise correlations between neighboring neurons of similar tuning, suggesting a mechanism of active decorrelation among nearby neurons. A recent review [62], that assessed experimental evidence for sparse coding, emphasized the particularly sparse response of layer 2/3 and pointed to the generally low firing activity in superficial layers compared with neurons from layer 4, which are considered to be a major input to layer 2/3. The functional characterization of PC responses is complemented by studies on physical connections between excitatory neurons. While inhibition was shown to act mostly locally, recurrent excitation spans larger distances [31]. Furthermore, neurons with positively correlated responses were found to also have an increased probability to maintain reciprocal connections [63]. More specifically, in [30] an increased connection probability was reported between neurons with similar orientation tuning and cell body distances larger than 500 *μ*m.

#### Cortical models propose similar network architectures and function

Based on the rich experimental data on cortical layer 2/3 both in vivo and in vitro, experimental neurobiologists and computational neuroscientists have proposed functional models of information processing in layer 2/3 that appear compatible with the architecture of the neural sheet model in this article. Douglas and Martin [29] as well as Lansner [35] sketched functional models of layer 2/3 that rely on local competitive circuits which communicate via associative excitatory links. The local competitive aspect of information processing in layer 2/3 was examined in detail by [50] and [52]. Finally, the architecture of the neural sheet model is highly reminiscent of the connectivity structures within a cortical column model of barrel cortex, as proposed in a recent review by Petersen and Crochet [64].

Especially cortical network models, which extend the idea of Bayesian confidence propagation neural networks (BCPNNs) [65], have been linked to probabilistic inference and statistical learning. In these networks, the mean activity of a small neuronal population (“cortical minicolumn”) is interpreted as the probability of a certain realization of a random variable. Different (disjoint) minicolumns form a “cortical hypercolumn” and compete in a winner-take-all manner to represent the possible values of a random variable. Excitatory synapses between hypercolumns realize associative memory function. BCPNN-type networks have been proposed as a model of human working memory [66]. Similar to the neural sheet model examined in this article, BCPNN-type networks are capable of developing abstract representations of input patterns [67] and forming associative memory [68], and support implementations with spiking neurons [69]. Furthermore, BCPNN-type networks have successfully been implemented with highly detailed neuron and synapse models [70]. A key difference between the neural sheet model and BCPNN-type networks is found in the local network architecture: In BCPNN-type networks, minicolumns are disjoint sets, i.e., each neuron participates in exactly one WTA circuit. In contrast, a neural sheet hosts a plethora of interwoven WTA subcircuits by virtue of the continuous nature of lateral inhibition. As a consequence, each network neuron can take part in multiple overlapping WTA subcircuits.

### Experimentally testable predictions

In our model, the correlation structure between stimulus features is reflected in the lateral connectivity of the network after learning. In the generative model, the plastic modification of lateral connections corresponds to reshaping the prior distribution *p*(***z*** ∣ ***θ***). Whether such correlation structure of features can be observed in the prior could be tested in an experiment that extends the setup of Berkes et al. [38]. There, it was observed in ferret V1 that the distribution of spontaneous activity (dark stimulus condition, identified with the prior *p*(***z*** ∣ ***θ***)) became increasingly similar with the average evoked activity (natural stimulus conditions, identified with the average posterior 〈*p* (***z*** ∣ ***y***, ***θ***)〉) during development. Our model predicts that a change in the correlation structure of environmental features should be mirrored in the generative prior and thus in the correlation structure of cortical spontaneous activity after learning.

In Fig 4, we have demonstrated synchronized perceptual switching between two modes of activity under ambiguous stimulus conditions. Such stochastic switching displays a characteristic property of sample-based representations when the posterior distribution is bimodal. Note that in our model, perceptual switching is a result of the learned prior *p*(***z*** ∣ ***θ***) that assigns very low probability to network states that seem inconsistent with previous experience. According to our model, the prior is subject to ongoing learning. Hence, for initial presentations of an ambiguous stimulus, the prediction is that network activity will alternate between response patterns that are consistent with the alternative stimulus interpretations rather than evoking a stable intermediate response (see Fig 4F). After sufficient learning time, however, it is expected that a distinct neural representation emerges for the previously ambiguous stimulus, on which the network activity settles.

### Related theoretical work and integration into larger networks

During the last decade, several theoretical studies examined how Bayesian computations could be performed and represented by spiking neural networks. In the following, we discuss how the neural sheet model relates to existing work with a focus on two key aspects: (1) The fundamental (and unanswered) question how probability distributions are encoded in neuronal activity patterns, and (2) the compatibility of the proposed sheet model, that focused on spatial aspects of distributed inference and learning, with spiking network models that addressed other aspects of Bayesian information processing, such as more complex causal relations or temporal integration. The two aspects are discussed separately.

#### Neural representations of Bayesian computation

The algorithms that underlie Bayesian computations in neural network models are diverse. However, regarding the representation of the arising posterior distributions *p* (***z*** ∣ ***y***, ***θ***) two general lines of research can be identified, namely sample-based codes and distributional codes. In sample-based codes (as employed by the neural sheet model) the observed network state is interpreted as an instantiation of one or more random variables ***z***. By observing the sequence ***z***(*t*) of network states over time, the distribution *p* (***z*** ∣ ***y***, ***θ***) is represented with increasing precision through the relative frequency of state occurrences [23, 24]. Examples of a direct (one-to-one) mapping between neurons and random variables are found in [38, 17, 25] and [20]. A conceptual separation between network neurons and the represented RVs was explored by [47] where only a subset of so-called principle neurons carry meaningful information for downstream populations. In [16], a spiking network model was proposed that enables a sample-based Bayesian interpretation of perceptual bistability based on the average activity of neuronal populations. The idea to rigorously separate neuronal spike patterns from the represented RVs was recently explored by [21], thereby allowing the simultaneous operation of multiple entangled sampling chains within a single network. In a different direction, recent theoretical work [18] has shown that the notion of “network states” can be extended to also cover entire population trajectories. On a general account, it has been emphasized [24] that sample-based codes offer several conceptual advantages, such as high representational flexibility, easy marginalization, natural emergence of response variability, and general suitability for learning.

Distributional codes provide a complementary (and at first glance, irreconcilable) neural representation of probability distributions. A characteristic property of distributional codes is the (almost) instantaneous representation of either the entire posterior distribution or, at least, pivotal statistical properties thereof (e.g. marginals or mean values). Just as the case for sampling networks, the exact inference algorithms implemented by distributional code network models are manifold. One line of research [11, 12, 14, 15], builds upon the belief propagation algorithm that aims to calculate the marginal posteriors 〈*z*_*k*_〉_*p* (***z*** ∣ ***y***, ***θ***)_ for all variables. This approach enables inference in complex graphical models, including temporal integration. However, since correlations among RVs in the posterior are not accommodated, the precise relative spike timing between different neurons carries no relevant information: what matters is the spike count in a given time interval, not the spike timing. A second line of research is established by (probabilistic) population codes (see e.g., [10, 13, 71]). These models aim to infer the posterior of a hidden (’true’) stimulus parameter (e.g., bar orientation) from the observed input activity in a known generative model. The inference process relies on integrating the spike count of many input neurons with known tuning properties, simultaneously. Neuronal trial-to-trial variability arises in this deterministic inference scheme solely from stochasticity in the spiking input.

Despite their different origins, sample-based codes and probabilistic population codes can provide mutually compatible interpretations, at least in some scenarios. Consider, for instance, a local column in the neural sheet model with multiple network neurons within the range of lateral inhibition. This local network is very similar to the competitive networks examined in [25]. Over the course of learning, each neuron will develop a tuning, e.g., a preferred bar orientation (cp. Fig 5 in [25]), such that the local network neurons will jointly cover the entire local input space. Due to the width of the likelihood distribution associated with each hidden cause, the response curves of roughly similarly tuned neurons will partially overlap. Formally, the resulting spike response of the local population to a given stimulus displays a sample-based code of a multinomial (mixture) posterior distribution. However, by knowing the tuning curves of each network neuron, the response of the sampling network could equally well be interpreted as a distributional code of the stimulus parameter (cp. Fig 2 in [71]). Conversely, the continuous hidden stimulus parameter in PPC models could always be mapped to a set of locally competing network neurons in a sampling sheet. Thus, it appears that, at least in some cases, sample-based codes and probabilistic population codes are mutually compatible, and further experimental research will be required to investigate spike response characteristics in more complex scenarios.

#### Integration into larger Bayesian spiking networks

In this work, we have employed a rather basic prior distribution *p*(***z*** ∣ ***θ***) for associative memory formation, namely a single-layer Boltzmann distribution. The theory of the neural sheet model, however, also supports more complex network structures such as deep learning architectures [39] that constitute one of the most powerful tools for unsupervised learning in machine learning theory. Such deep network architectures would add hierarchical information exchange to the system (e.g., top-down or contextual information). Furthermore, arbitrary Bayesian networks, i.e., directed graphical models, could likely be used for the prior distribution as long as the graphical model features the local lateral inhibition network motif (mutually exclusive activity of hidden variables with shared input). Several spiking network implementations for sample-based inference in general graphical models can be found in [47]. Notably, these implementations overcome the constraint of symmetric recurrent connections in the network. An asymmetric recurrent connectivity structure is also a salient property of temporal models. To endow the spatial model with the ability of temporal integration, it would therefore be intriguing to combine the proposed sheet model with the Hidden Markov Model network implementation by [20] or the neural particle filtering approach by [22]. In a different research direction, we observed in computer simulations that relaxing the assumption of strong lateral inhibition still leads to reasonable learning results in many scenarios. This indicates that even soft inhibition could be sufficient to govern the emergence of probabilistic local experts—an important property for network models that feature a high neuron density. However, our current theory does not provide a proper interpretation of the resulting network response since the arising coactivation of neighboring neurons likely demands a generative model that supports soft explaining away. Finally, it would be interesting to combine the fully self-organized network model with reinforcement learning signals from the environment (e.g., via top-down feedback or third-factor plasticity rules). This could endow the spiking network with the ability of Bayesian decision making and action selection.

### Operation paradigm for novel computing platforms

Beyond neuroscience, the proposed neural sheet model displays an intriguing design principle for neuromorphic architectures [72]. Neuromorphic systems rely on rigorous parallelization of information processing by implementing physical models of neurons and synapses in microscale electronic circuitry [73]. A key intention behind the development of these systems is the construction of fault tolerant, self-organized computing devices that overcome the traditional strictly serial and deterministic design of von Neumann architectures.

The proposed neural sheet model appears to be ideally suited for neuromorphic implementations: information exchange is fully asynchronous without a central clock; the emergent sparse spike code reduces the load on the interneuronal bus system; and the local confinement of lateral inhibition facilitates compact mapping and efficient routing. Furthermore, idealized modeling assumptions, such as stochastic neurons, rectangular PSP shapes or tailored inhibitory connections, could likely be accounted for in engineered systems.

One of the main challenges in neuromorphic engineering lies in the required high-density integration of plastic synapses [74]. On the way to a principled solution, material scientists have made great progress in recent years in using memristors as plastic synapses [75, 76]. Memristors [77, 78] are novel nanoscale materials that adapt their electrical conductance based on the history of the current and voltage flux through the device. Thus, memristors are expected to accommodate both synaptic transmission and synaptic plasticity without the need of extensive supporting circuitry. Experimental [79, 80] and theoretical [81, 82] studies have explored how the plasticity dynamics of memristors can be utilized in spiking neural networks. In particular, it was shown in [83] and [84] that conductance changes in certain memristive materials appear compatible with (variants of) the weight dependent plasticity rule Eq (12). Therefore, we expect that the proposed neural sheet model can provide a promising paradigm for the efficient operation of neuromorphic hardware as massively parallel computing devices for probabilistic inference and self-organized learning.

### Conclusion

We have proposed a spiking neural sheet model for sample-based probabilistic inference and self-organized learning. The spatially structured network combines aspects of local competitive learning and large-scale associative memory formation under a unified Bayesian account. Using machine learning theory, we have shown how the spike response of the neural sheet can be interpreted as an ongoing sampling process from a Bayesian posterior distribution, and how local neural plasticity can accomplish network-wide statistical model optimization. The network structure of the neural sheet bears resemblance to salient connectivity motifs observed experimentally in cortical microcircuits. Therefore, we believe that the theoretical findings presented in this article can contribute to the development of targeted experiments on synaptic plasticity and neural coding in the mammalian brain.

## Methods

In the following, we provide the full definition and derivation of the neural sheet model for inference and unsupervised learning. We first describe the probabilistic model and show that the spiking network model can sample from the posterior distribution. Then we derive parameter updates for unsupervised model optimization and link them to plasticity rules for sample-based online learning. The heuristic recurrent plasticity rule and a brief discussion on the interacting time scales are presented in separate subsections. Finally, we provide details to the computer simulations and figures.

### Generative model

#### Definition of variables

We introduce a generative model

$$p\;(y,z\;|\;\theta )=p_{}\;(y\;|\;z,\;\theta )\cdot p\;(z\;|\;\theta )$$ (20)

over *N* *observed variables* *y*_1_, .., *y*_*N*_, subsumed in the vector ***y***, and *K* *latent variables* *z*_1_, .., *z*_*K*_, subsumed in the vector ***z***. The latent variables are binary, *z*_*k*_ ∈ {0,1}, and said to be “active” iff *z*_*k*_ = 1. The possible values of the observed variables *y*_*i*_ depend on the employed likelihood model. The full model is governed by parameters $\theta =(V,\;V_{0},\;\hat{W},\;\hat{b})$ with real-valued *K* × *N* *afferent weight matrix* ***V***, *N*-dimensional *default vector* ***V***_0_ (which will be a constant during learning), *K* × *K* *recurrent weight matrix* $\hat{W}$, and *K*-dimensional *bias vector* $\hat{b}$. Furthermore, each latent variable *z*_*k*_ is *connected* to a subset of the input variables (its *afferent field*) which is described by an index set ℐ_k_ ⊆ {1, 2, .., *N*}. Likewise, we define the *projection field* for each input variable *y*_*i*_ as the index set ${𝒫}_{i}\;=\;\left\{k\;|\;i\in \;{𝓘}_{k}\right\}$. We refer to all-but-one variables of a vector by the shorthand notation ***z***_\*k*_ ≔ (*z*_1_, .., *z*_*k*−1_, *z*_*k* + 1_, .., *z*_*K*_).

#### Generative Model: Likelihood

Provided the state of the hidden units ***z***, the inputs are defined to be independent (local “Naive Bayes”):

$$p_{}\;(y\;|\;z,\;\theta )=\prod_{i=1}^{N}p_{}\;(y_{i}\;|\;z,\;\theta ).$$ (21)

For each *y*_*i*_, the prior will ensure that at most one hidden unit *z*_*k*_ in ${𝒫}_{i}$ is active. We assume that, if present, this single active unit *z*_*k*_ governs the distribution of *y*_*i*_. In case all hidden variables in ${𝒫}_{i}$ are 0, a “default hypothesis” is used. The resulting input distribution is assumed to be in the natural exponential family. For instance, Bernoulli, Poisson, or Gaussian distributions are in this class. In natural exponential family form, *p* (*y*_*i*_ ∣ ***z***, ***θ***) reads:

$$p\;\left(y_{i}|z_{k}=0,\;\forall \;k\in {𝒫}_{i},\;\theta \right)=h_{i}\;\left(y_{i}\right)\;e^{V_{{}_{0i}}\;y_{i}-A_{0i}}$$ (22)

$$p\;\left(y_{i}|z_{k}=1,k\in {𝒫}_{i},\theta \right)=h_{i}\;\left(y_{i}\right)\;e^{\left(V_{ki}+V_{0i}\right)\;y_{i}-\;\left(A_{ki}+A_{0i}\right)}$$ (23)

where *h*_*i*_(*y*_*i*_) denotes the base measure, and the normalization constants *A*_*ki*_ and *A*_0*i*_ depend on the parameters *V*_*ki*_ and *V*_0*i*_. In anticipation of (25), the parameters in (23) were written as (*V*_*ki*_ + *V*_0*i*_), i.e. relative to the default hypothesis. We adopt the convention that *V*_*ki*_ = 0 for $k\;\notin \;{𝒫}_{i}$ to obtain a closed form expression for (22) and (23):

$$p\;\left(y_{i}|z,\theta \right)=h_{i}\;\left(y_{i}\right)\;e^{V_{0i}\;y_{i}-A_{0i}}\underset{k\in {𝒫}_{i}}{{\prod \;}^{}}{\left[e^{V_{ki}\;y_{i}-A_{ki}}\right]}^{Z_{k}}$$ (24)

$$=h_{i}\left(y_{i}\right)\;e^{V_{0i}\;y_{i}-A_{0i}}\text{exp}\;\left[\sum_{k=1}^{K}z_{k}V_{ki}y_{i}-z_{k}A_{ki}\right]$$ (25)

By combining (21) and (25) we obtain the full likelihood

$$p\;(y\;|\;z,\;\theta )=h\left(y\right)\;\exp[z^{⊺}Vy-z^{⊺}A]$$ (26)

with ***A*** = (*A*_1_, .., *A*_*K*_)^⊺^ and $A_{k}=\sum_{i=1}^{N}A_{{}_{ki}}$. Here we use the short hands ***z*** ^⊺^ ***V y*** = ∑_k_∑_i_ *z_k_* *V_ki_y_i_* and ***z*** ^⊺^ ***A*** = ∑_*k*_ *z_k_A_k_*. The function *h*(***y***)≔ Π_*i*_ *h_i_*(*y_i_*) *e* ^*V*_0*i*_*y*_*i*_−*A*_0*i*_^ comprises only terms that do not depend on ***z***, and hence, it will play no role in the inference. In Results, we employed a Bernoulli likelihood model:

$$p\;\left(y_{i}=1|z_{k}=0,\;\forall \;k\in {𝒫}_{i},\;\theta \right)=\pi_{0i}\;\;,\;p\;\left(y_{i}=1|z_{k}=1,\;k\in {𝒫}_{i},\;\theta \right)=\pi_{ki}$$ (27)

or in closed form:

$$p\;\left(y_{i}|z,\;\theta \right)=\pi_{0i}^{y_{i}}{\left(1-\pi_{0i}\right)}^{1-y_{i}}\;{\prod_{k}[\frac{\pi_{ki}^{y_{i}}{\left(1-\pi_{ki}\right)}^{1-y_{i}}}{\pi_{0i}^{y_{i}}{\left(1-\pi_{0i}\right)}^{1-y_{i}}}]}^{zk}.$$ (28)

By rewriting (28) in the exponential family form (25), we identify

$$\begin{array}{ll} V_{ki}=\text{log}\frac{\pi_{ki}}{1-\pi_{ki}}-V_{0i}, & A_{ki}=\text{log}\left(1+e^{V_{ki}+V_{0i}}\right)-A_{0i},\\ V_{0i}=\text{log}\frac{\pi_{0i}}{1-\pi_{0i}}, & A_{0i}=\text{log}\left(1+e^{V_{0i}}\right). \end{array}$$ (29)

In particular, the cluster centers can be recovered via *π*_*ki*_ = *σ*(*V*_*ki*_ + *V*_0*i*_).

Likewise, Poisson and Gaussian distributions can be written in the exponential family form (25). This extends the input domain to *y*_*i*_ ∈ ℕ and *y*_*i*_ ∈ ℝ respectively. For a Poisson model with expected values *λ*_0*i*_ and *λ*_*ki*_ (for the default hypothesis and the hidden causes resp.),

$$p_{}(y_{i}\;|\;z_{k}=0,\;\forall \;k\in {𝒫}_{i},\;\theta )={\left(y_{i}!\right)}^{-1}\lambda_{0i}^{y_{i}}e^{-\lambda_{0i}}\;\;,\;p_{}(y_{i}\;|\;z_{k}=1,\;k\in {𝒫}_{i},\;\theta )={\left(y_{i}!\right)}^{-1}\lambda_{ki}^{y_{i}}e^{-\lambda_{ki}},$$ (30)

we obtain

$$\begin{array}{ll} V_{ki}=\text{log}\;\left(\lambda_{ki}\right)-V_{0i}, & A_{ki}=\text{exp}\;\left(V_{ki}+V_{0i}\right)-A_{0i},\\ V_{0i}=\text{log}\;\left(\lambda_{0i}\right), & A_{0i}=\text{exp}\;\left(V_{0i}\right). \end{array}$$ (31)

For Gaussians with centers *μ*_0*i*_ and *μ*_*ki*_ and fixed variance *σ* ^2^,

$$p\;(y_{i}\;|\;z_{k}=0,\;\forall \;k\in {𝒫}_{i},\;\theta )=𝒩\;\left(y_{i};\;\mu_{0i},\sigma^{2}\right)\;\;,\;p\;(y_{i}\;|\;z_{k}=1,\;k\in {𝒫}_{i},\;\theta )=𝒩\;\left(y_{i};\;\mu_{ki},\sigma^{2}\right),$$ (32)

we obtain

$$\begin{array}{ll} V_{ki}=\frac{\mu_{ki}}{\sigma^{2}}-V_{0i}, & A_{ki}=\frac{\sigma^{2}}{2}{\left(V_{ki}+V_{0i}\right)}^{2}-A_{0i},\\ V_{0i}=\frac{\mu_{0i}}{\sigma^{2}}, & A_{0i}=\frac{\sigma^{2}}{2}V_{0i}^{2}. \end{array}$$ (33)

#### Generative model: Prior

The prior *p*(***z*** ∣ ***θ***) guarantees that no two (or more) hidden units *z*_*k*_, *z*_*j*_ with overlapping input fields, ℐ_*k*_ ∩ℐ_*j*_ ≠ ∅, are active simultaneously, i.e., it ensures that at most one unit *z*_*k*_ generates each input variable *y*_*i*_ at the same time. For this work, we choose a Boltzmann machine prior which can introduce dependencies between units with non-overlapping receptive fields through symmetric parameters ${\hat{W}}_{kj}^{\text{exc}}={\hat{W}}_{jk}^{\text{exc}}$. Furthermore each variable *z*_*k*_ has a bias value ${\hat{b}}_{k}$ which directly affects its prior probability of activity:

$$p\;(z\;|\;\theta )=\frac{1}{Z}\text{exp}\;\left[\frac{1}{2}z^{⊺}\;{\hat{W}}^{\text{exc}}z+z^{⊺}\hat{b}\right]\;\cdot \;\overset{K}{\underset{k=1}{\prod }}\overset{K}{\underset{j=1}{\prod }}{\left(\delta_{{ℐ}_{k}\cap^{}{ℐ}_{j},\;\;\emptyset }\right)}^{z_{k}\cdot z_{j}},$$ (34)

with *δ* denoting the Kronecker delta and 0^0^ ≔ 1. The double-product factor ensures, that the assumptions on ***z*** made in the likelihood (26) are satisfied, and can be approximated with arbitrary precision by using strong inhibitory weights ${\hat{W}}_{jk}^{\text{inh}}$

$${ℐ}_{k}\cap^{}{ℐ}_{j}\neq \emptyset \Rightarrow {\hat{W}}_{kj}^{inh}\to -\infty \;\text{for}\;k\neq j,$$ (35)

and setting $\prod_{k=1}^{K}\prod_{j=1}^{K}{\left(\delta_{{ℐ}_{k}\cap {ℐ}_{j},\;\emptyset }\right)}^{z_{k}\cdot z_{j}}=\text{exp}[\frac{1}{2}z^{⊺}{\hat{W}}^{\text{inh}}z]$. Note that the range of strong inhibition could also extend beyond the minimal required range (35). For notational brevity, we subsume the excitatory and inhibitory recurrent weight matrices in a single matrix $\hat{W}={\hat{W}}^{\text{exc}}+{\hat{W}}^{\text{inh}}$ for the derivation. $\hat{W}$ is symmetric ($\hat{W}={\hat{W}}^{⊺})$ and has zero diagonal (${\hat{W}}_{kk}=0$). Using this notation, the prior simply reads

$$p\;(z\;|\;\theta )=\frac{1}{Z}\exp[\frac{1}{2}z^{⊺}\hat{W}z+z^{⊺}\hat{b}].$$ (36)

### Inference in the generative model (Corollary 1)

By applying Bayes rule *p* (***z*** ∣ ***y***, ***θ***) ∝ *p*(***z*** ∣ ***θ***) ⋅ *p* (***y*** ∣ ***z***, ***θ***) on Eqs (36) and (26) we obtain the posterior in closed form:

$$p\;(z\;|\;y,\;\theta )=\exp[\frac{1}{2}z^{⊺}\hat{W}z+z^{⊺}Vy+z^{⊺}\;(\hat{b}-A)]\;/\;\text{Norm}.$$ (37)

where the normalization sums the exponential over all possible states of the posterior. To establish the link to the neural sampling theory via the sufficient conditions (5), we solve (37) for the logit of a single unit *z*_*k*_:

$$u_{k}\overset{!}{=}\log\frac{p_{}\;(z_{k}=1\;|\;z_{\backslash k},\;y,\;\theta )}{p_{}\;(z_{k}=0\;|\;z_{\backslash k},\;y,\;\theta )}=\log\frac{p_{}\;(z_{k}=1,\;z_{\backslash k}\;|\;y,\;\theta )}{p_{}\;(z_{k}=0,\;z_{\backslash k}\;|\;y,\;\theta )}$$ (38)

$$=\log\frac{\exp[\frac{1}{2}\sum_{j\neq k}\left(1\cdot {\hat{W}}_{kj}z_{j}+z_{j}{\hat{W}}_{jk}\cdot 1\right)+\sum_{i}\left(1\cdot V_{ki}y_{i}\right)+1\cdot \left({\hat{b}}_{k}-A_{k}\right)]}{\exp[\frac{1}{2}\sum_{j\neq k}\left(0\cdot {\hat{W}}_{kj}z_{j}+z_{j}{\hat{W}}_{jk}\cdot 0\right)+\sum_{i}\left(0\cdot V_{ki}y_{i}\right)+0\cdot \left({\hat{b}}_{k}-A_{k}\right)]}$$ (39)

$$+\log\frac{\exp[\frac{1}{2}\sum_{j,l\neq k}z_{j}{\hat{W}}_{jl}z_{l}+\sum_{j\neq k}\sum_{i}\left(z_{j}V_{ji}y_{i}\right)+\sum_{j\neq k}z_{j}\cdot \left({\hat{b}}_{j}-A_{j}\right)]}{\exp[\frac{1}{2}\sum_{j,l\neq k}z_{j}{\hat{W}}_{jl}z_{l}+\sum_{j\neq k}\sum_{i}\left(z_{j}V_{ji}y_{i}\right)+\sum_{j\neq k}z_{j}\cdot \left({\hat{b}}_{j}-A_{j}\right)]}$$ (40)

$$=\log\frac{\exp[\sum_{j\neq k}{\hat{W}}_{kj}z_{j}+\sum_{i}V_{ki}y_{i}+\left({\hat{b}}_{k}-A_{k}\right)]}{\exp[0]}+\log1$$ (41)

$$=\sum_{j=1}^{K}{\hat{W}}_{kj}z_{j}+\sum_{i=1}^{N}V_{ki}y_{i}+\left({\hat{b}}_{k}-A_{k}\right).$$ (42)

Here we made use of the symmetry of $\hat{W}$ and ${\hat{W}}_{kk}=0$. Hence, we can map the neuronal membrane potential (3) to the parameters of the Bernoulli likelihood model. We find ${\hat{W}}_{kj}={\hat{W}}_{kj}^{\text{exc}}+{\hat{W}}_{kj}^{\text{inh}}=W_{kj}^{\text{exc}}+W_{kj}^{\text{inh}}$ for recurrent weights, $V_{ki}=\text{log}\frac{\pi_{ki}}{1-\pi_{ki}}-\text{log}\frac{\pi_{0i}}{1-\pi_{0i}}$ for afferent weights according to Eq (29), and $b_{k}={\hat{b}}_{k}-A_{k}$ for excitabilities, with *A*_*k*_ = ∑_*i*_ *A*_*ki*_ given by Eq (29). This proves Corollary 1.

### Model optimization via Generalized Expectation Maximization

In the presence of plastic input synapses *V*_*ki*_, the terms *A*_*k*_ change over time and, in a spiking network implementation, add to the intrinsic excitability of the cells. Arguably, the information required for calculating *A*_*k*_ is not locally available to the neurons since *A*_*k*_ depends on all afferent synaptic efficacies *V*_*ki*_. We follow the approach of [36] who showed that homeostatic intrinsic plasticity can enable a spiking network to account for time varying *A*_*k*_’s. More precisely, the interplay of homeostatic intrinsic plasticity and synaptic plasticity can be understood in the generalized Expectation Maximization framework, and a spiking network can implement a variational posterior distribution *q*(***z*** ∣ ***y***) which maintains a long-term average target activity. In close analogy to the derivation in [36], we transfer this approach to the spatially extended sheet model in the following.

We impose a posterior constraint on the latent variables ***z*** and investigate learning in the generalized online EM framework. The EM decomposition [85] reads

$$ℱ\;\left(\theta ,q\;(z|y)\right)=ℒ\left(\theta \right)-{\langle D_{\text{KL}}(\;q(z|y)\;\|\;p(z\;|\;y,\;\theta )\;)\rangle }_{p^{*}\left(y\right)}\;\to \text{E-step},$$ (43)

$$={\langle \log p\;(y,z\;|\;\theta )\rangle }_{p^{*}\left(y\right)q\left(z|y\right)}+{\langle H\left(q\left(z|y\right)\right)\rangle }_{p^{*}\left(y\right)}\;\to \text{M-step},$$ (44)

with the log-likelihood ℒ(***θ***) = 〈log *p* (***y*** ∣ ***θ***)〉_p*(***y***)_ of the input under the model, the Kullback-Leibler divergence *D*_KL_(⋅∣∣⋅), and the entropy *H*(⋅). The decomposition holds for any probability distribution *q*, and *q*(***z*** ∣ ***y***) defines a variational posterior for every input state. For this work, we constrain *q* to a class of “homeostatic” distributions, q ∈ 𝓠, such that each variable *z*_*k*_ maintains a long-term average activity *m*_*k*_,

$$𝓠=\{q:{\langle z_{k}\rangle }_{p^{*}\left(y\right)q\left(z|y\right)}=m_{k}\;\text{for all}\;k=1,..,K\}.$$ (45)

The desired target activations ***m*** = (*m*_1_, .., *m*_*K*_) are assumed to be compatible with the inhibition structure (35), e.g. by choosing *m*_*k*_ sufficiently small.

#### E-step: Homeostatic intrinsic plasticity

During the E-step (43), we seek the distribution $q^{*}\;\in \;𝒬$ that minimizes the Kullback-Leibler divergence to the model posterior *p* (***z*** ∣ ***y***, ***θ***), and thus, maximizes the lower bound ℱ on the likelihood ℒ. This constrained optimization problem can be solved with Lagrange multipliers. We examine the Lagrange function

$$\Lambda \left(q\right)=\langle D_{\text{KL}}(\;q\left(z^{\prime }\;|\;y^{\prime }\right)\;||\;p_{}(z^{\prime }\;|\;y^{\prime },\;\theta )\;{)\rangle }_{p^{*}\left(y^{\prime }\right)}-\sum_{k=1}^{K}\beta_{k}\;({\langle z_{k}^{\prime }\rangle }_{q\left(z^{\prime }\;|\;y^{\prime }\right)\;p^{*}\left(y^{\prime }\right)}-m_{k})-\lambda \;({\langle 1\rangle }_{q(z^{\prime }\;|\;y^{\prime })}-1)$$ (46)

where the apostrophes indicate that ***y***′ and ***z***′ are summation variables, and *β*_*k*_ are the Lagrange multipliers for the *K* constraints. The additional Lagrange multiplier *λ* ensures correct normalization of *q*. The root of the derivative with respect to *q*(***z*** ∣ ***y***), with any particular choice of ***z*** and ***y***, fulfills $\partial_{q(z\;|\;y)}\Lambda (q)=p^{*}(y)\;\left[\text{log}\left(q(z\;|\;y)\;/\;p_{}\left(z\;|\;y,\;\theta \right)\right)+1-\lambda -\sum_{k}\beta_{k}\;z_{k}\right]\;\overset{!}{=}0\;$ and, thus, the optimal solution q* has the form

$$q^{*}\left(z\;|\;y\right)=p_{}(z\;|\;y,\;\theta )\;\exp[\lambda -1+\sum_{k}\beta_{k}\;z_{k}]\;\propto \exp[\frac{1}{2}z^{⊺}\hat{W}z+z^{⊺}Vy+z^{⊺}(\underset{≔\;b\;=\;(b_{1},..,b_{K})}{\underset{︸}{\hat{b}-A+\beta }})]\;\;.$$ (47)

Note that the variational distribution in (47) is already correctly normalized through the free constant exp(*λ*−1).

However, the optimal multipliers ***β*** = (*β*_1_, .., *β*_*K*_) are still to be determined. Analogous to [36, 37], gradient ascent on the dual function

$$\partial_{\beta_{k}}\Psi \left(\beta \right)=\partial_{\beta_{k}}\;\beta^{T}\;m-{\langle \;\partial_{\beta_{k}}\;\log\sum_{z}p_{}\;(z\;|\;y,\;\theta )\;\text{exp}\left(\beta^{T}\;z\right)\;\rangle }_{p^{*}\left(y\right)}$$ (48)

$$=m_{k}-{\langle z_{k}\rangle }_{p^{*}\left(y\right)q\left(z|y\right)}$$ (49)

yields an iterative update rule to determine the optimal Lagrange multipliers *β*_*k*_ in q* for the E-step (43). During the E-step, the synaptic weights *V*_*ki*_ remain constant (synaptic weight updates are the M-step). Thus, optimizing *β*_*k*_ is equivalent to optimizing $b_{k}≔{\hat{b}}_{k}-A_{k}+\beta_{k}$ since *b*_*k*_ and *β*_*k*_ differ only by an additive constant. In particular, the update rule

$$\partial_{b_{k}}\Psi =\partial_{\beta_{k}}\Psi =m_{k}-{\langle z_{k}\rangle }_{p^{*}\left(y\right)q\left(z|y\right)}$$ (50)

remains unchanged and describes a form of homeostatic plasticity. It compares the average activation 〈*z*_*k*_〉_p*(***y***)*q*(***z***∣***y***)_ with the target activation *m*_*k*_ and adapts the intrinsic excitability accordingly: When the average activity 〈*z*_*k*_〉 is too low, the excitability *b*_*k*_ will be increased; if the activity is too high, the excitability will be reduced. Importantly, the homeostatic rule (50) requires only local information and “overwrites” the non-local terms *A*_*k*_ in (47) and in *b*_*k*_.

#### M-step in *V*_*ki*_: Weight-dependent plasticity of afferent weights

During the M-step (44), we perform gradient ascent on 〈log *p* (***y***, ***z*** ∣ ***θ***)〉_p*(***y***)*q*(***z***∣***y***)_ with respect to the parameters ***θ***. This increases the lower bound ℱ on the likelihood ℒ(***θ***) since the entropy 〈*H*(*q*(***z***∣***y***))〉_p*(***y***)_ does not depend on ***θ***. For the afferent weights ***V***, the derivative of the log-joint models (26) × (36) reads

$$\partial_{V_{ki}}𝓕=\partial_{V_{ki}}{\langle \;\log p\;(y,z\;|\;\theta )\rangle }_{p^{*}\left(y\right)q\left(z|y\right)}={\langle z_{k}\cdot \left(y_{i}-\partial_{V_{ki}}A_{ki}\right)\;\rangle }_{p^{*}\left(y\right)q\left(z|y\right)}.$$ (51)

For a Bernoulli likelihood distribution, we obtain from Eq (29) that ∂_*V*_*ki*__ *A*_*ki*_ = *σ*(*V*_*ki*_ + *V*_0*i*_) = *π*_*ki*_ and hence:

$$\partial_{V_{ki}}𝓕={\langle \;z_{k}\cdot \left(y_{i}-\sigma \left(V_{ki}+V_{0i}\right)\right)\;\rangle }_{p^{*}\left(y\right)q\left(z|y\right)}.$$ (52)

The update rule Eq (52) only depends on local information, namely pre-(*y*_*i*_) and post-(*z*_*k*_) synaptic activity and the current synaptic weight *V*_*ki*_. The same holds true for Poisson distributions with ∂_*V*_*ki*__ *A*_*ki*_ = exp(*V*_*ki*_ + *V*_0*i*_) and Gaussian distributions with ∂_*V*_*ki*__ *A*_*ki*_ = *σ* ^2^ ⋅ (*V*_*ki*_ + *V*_0*i*_). Intuitively, since ∂_*V*_*ki*__ *A*_*ki*_ = 〈*y*_*i*_〉_*p* (*y*_*i*_ ∣ *z*_*k*_ = 1, ***θ***)_ in any natural exponential family, the plasticity rule (51) compares the true input value *y*_*i*_ ∼ p*(***y***) with the current expectation of the probabilistic model whenever *z*_*k*_ is active.

#### M-step in ${\hat{W}}_{kj}^{\text{exc}}$: Wake-sleep plasticity of recurrent weights

Similarly, we can examine the derivative of ℱ with respect to the recurrent weights $\hat{W}$ and biases $\hat{b}$ in the prior:

$$\partial_{{\hat{W}}_{kj}}{\langle \;\log p\;(y,z\;|\;\theta )\rangle }_{p^{*}\left(y\right)q\left(z|y\right)}={\langle z_{k}\;z_{j}\rangle }_{p^{*}\left(y\right)q\left(z|y\right)}-{\langle z_{k}\;z_{j}\;\rangle }_{p(z\;|\;\theta )}$$ (53)

$$\partial_{{\hat{b}}_{k}}{\langle \log p\;(y,z\;|\;\theta )\rangle }_{p^{*}\left(y\right)q\left(z|y\right)}={\langle z_{k}\rangle }_{p^{*}\left(y\right)q\left(z|y\right)}-{\langle \;z_{k}\;\rangle }_{p(z\;|\;\theta )}$$ (54)

These update rules compare expected values from the variational posterior with expected values from the model prior, i.e., they are a variant of the wake-sleep algorithm. In the neural sheet model, we will apply recurrent learning only to lateral excitatory connections $W_{kj}^{\text{exc}}$ and keep lateral inhibitory connections $W_{kj}^{\text{inh}}$ fixed since lateral inhibition is the foundation for local synaptic plasticity rules of the afferent connections *V*_*ki*_.

#### Plasticity rules for sample-based online learning (Corollary 2)

The above learning scheme revealed local update rules for unsupervised model optimization via Generalized Expectation Maximization. The derived algorithm relies on three ingredients:

1. the variational posterior distribution *q*(***z*** ∣ ***y***) in Eq (47),
2. the homeostatic update rule (50) to solve the E-step, and
3. the synaptic update rule (52) to solve the M-step.

*Ingredient 1:* By applying the sufficient condition (5) on the homeostatic posterior *q*(***z*** ∣ ***y***), we find that the network can sample from *q*(***z*** ∣ ***y***) when we use the same form of the membrane potential *u*_*k*_, but with $b_{k}≔{\hat{b}}_{k}-A_{k}+\beta_{k}$ (instead of $b_{k}={\hat{b}}_{k}-A_{k}$ for sampling from the model posterior *p* (***z*** ∣ ***y***, ***θ***)). This establishes an equivalent to Corollary 1 for the variational posterior.

*Ingredient 2:* A sample-based online approximation of Eq (50) is established by

$$\frac{\partial }{\partial t}\;b_{k}=\eta_{b}\cdot \left(m_{k}-z_{k}\left(t\right)\right)\;\;,$$ (55)

with *η*_*b*_ denoting a small learning rate. This homeostatic intrinsic plasticity rule approximates the expected values 〈*z*_*k*_〉_p*(***y***)*q*(***z*** ∣ ***y***)_ in Eq (50) through samples ***z*** ∼ *q*(***z*** ∣ ***y***) in response to the input ***y*** ∼ p*(***y***). The required samples ***z*** ∼ *q*(***z*** ∣ ***y***) ⋅ p*(***y***) are naturally provided by the network in response to presented input. The homeostatic plasticity rule Eq (55) uses only locally available information. When homeostatic plasticity has converged, i.e., when the average update $\langle \frac{\partial }{\partial t}\;b_{k}\rangle =0$ vanishes for all neurons, the network implements the variational posterior distribution Eq (47) with optimal multipliers *β*_*k*_, i.e., the network solves the E-step by sampling from from q*(***z*** ∣ ***y***). Due to the non-infinitesimal learning rate *η*_*b*_, this equilibrium is subject to small stochastic fluctuations.

*Ingredient 3:* A sample-based online approximation of Eq (52) is established by

$$\frac{\partial }{\partial t}\;V_{ki}=\eta_{V}\cdot z_{k}\left(t\right)\cdot (y_{i}\left(t\right)-\sigma \left(V_{ki}+V_{0i}\right)),$$ (56)

with a small learning rate *η*_*V*_. This synaptic plasticity rule approximates the gradient in Eq (52) from samples ***y*** ∼ p*(***y***) and the evoked response ***z*** ∼ *q*(***z*** ∣ ***y***). The required samples ***z*** ∼ *q*(***z*** ∣ *y*) ⋅ p*(***y***) are provided by the network if the homeostatic posterior *q*(***z*** ∣ ***y***) is correctly implemented in the E-step. Hence, it must be ensured that homeostatic intrinsic plasticity acts on significantly faster time scales than synaptic plasticity. This can be achieved by separating the time scales of intrinsic and synaptic plasticity via the learning rates *η*_*b*_ and *η*_*V*_, such that homeostatic intrinsic plasticity can react quickly to changes in the synaptic weights (see also *Interaction of time scales* below).

Since Eq (55) approximates the E-step, and Eq (56) approximates the M-step, the joint application of these rules approximates the previously described Generalized Expectation Maximization algorithm. This proves Corollary 2.

In addition, theoretically optimal learning of recurrent connections can be realized by sample-based implementations of Eqs (53) and (54). This gives rise to the plasticity rule Eq (16). The LTD term $\phi_{kj}^{\text{opt}}\left({\hat{W}}^{\text{exc}},{\hat{W}}^{\text{inh}},\hat{b}\right)={\langle \;z_{k}\;z_{j}\;\rangle }_{p\left(z\;|\;\theta \right)}$ can be determined by using an independent prior sampler, thereby giving rise to a sample-based sleep phase. In computer simulations with wake-sleep learning, we used such a prior sampler. This sampler maintained independent parameters $\hat{b}$ which were updated according to a sample-based approximation of Eq (54), i.e., according to the difference of samples from the variational posterior and the prior. Since it is not known if (or how) the LTD term $\phi_{kj}^{\text{opt}}\left({\hat{W}}^{\text{exc}},{\hat{W}}^{\text{inh}},\hat{b}\right)$ can be calculated by a single spiking network, we did not include recurrent plasticity in Corollary 2. Nevertheless, algorithmically the theory supports concurrent learning of input synapses and recurrent synapses.

### Approximate plasticity rule for recurrent synapses

As shown above, theoretically optimal learning of recurrent weights $W_{kj}^{\text{exc}}$ is challenging in a spiking network since the model expectations in Eq (53) are not directly available from the network response and demand an independent sleep phase. Therefore, we investigated to what extent the simple local plasticity rule (17) could entail similar weight configurations as the theoretically exact wake-sleep rule. In the following, we describe how the simple rule was obtained.

For a given learning problem, we first performed wake-sleep learning to obtain optimized weights $W_{kj}^{\text{opt}}$, along with covariances *c*_*kj*_: = 〈(*z*_*k*_−〈*z*_*k*_〉) ⋅ (*z*_*j*_−〈*z*_*j*_〉)〉_p*(***y***)*q*(***z*** ∣ ***y***)_ that led to these weights. Then, we fit a function *W*(*c*_*kj*_) to the data. For constructing the local plasticity rule, we use that *m*_*k*_ = 〈*z*_*k*_〉_p*(***y***)*q*(***z*** ∣ ***y***)_ due to homeostatic intrinsic plasticity, i.e.,

$$c_{kj}={\langle z_{k}\cdot z_{j}\rangle }_{p^{*}\left(y\right)q\left(z\;|\;y\right)}-m_{k}\cdot m_{j}\;\;.$$ (57)

Importantly, the information 〈*z*_*k*_ ⋅ *z*_*j*_〉_p*(***y***)*q*(***z*** ∣ ***y***)_ on the pre-post spike response during inference is locally available to a synapse. The following weight-dependent plasticity rule for $W_{kj}^{\text{exc}}$ then has a fixed point at $W_{kj}^{\text{exc}}=W(c_{kj})\approx W_{kj}^{\text{opt}}(c_{kj})$:

$$\frac{\partial }{\partial t}W_{kj}^{\text{exc}}=\eta_{W}\cdot \;\left[z_{k}\cdot z_{j}-\left(m_{k}\cdot m_{j}+W^{-1}\left(W_{kj}^{\text{exc}}\right)\right)\right]$$ (58)

with *W* ^−1^ denoting the inverse function of *W*(*c*_*kj*_). This follows directly from inspecting

$$0\overset{!}{=}{\langle \frac{\partial }{\partial t}W_{kj}^{\text{exc}}\rangle }_{p^{*}\left(y\right)q(z\;|\;y)}={\langle z_{k}\cdot z_{j}\rangle }_{p^{*}\left(y\right)q(z\;|\;y)}-m_{k}\cdot m_{j}-W^{-1}\left(W_{kj}^{\text{exc}}\right)$$ (59)

and solving for $W_{kj}^{\text{exc}}$. For this work, we assumed the following functional form of *W*(*c*_*kj*_):

$$W\left(c_{kj}\right)=\frac{W^{\text{max}}}{\pi /2}\arctan\left(\gamma \cdot c_{kj}\right)$$ (60)

with two free parameters *W* ^max^ and *γ*. This function turned out to match the data points $\left(W_{kj}^{\text{opt}},c_{kj}\right)$, as obtained from wake-sleep learning, well, and results in the following heuristic plasticity rule:

$$\frac{\partial }{\partial t}W_{kj}^{\text{exc}}=\eta_{W}\;\cdot \;\left[z_{k}\cdot z_{j}-\left(m_{k}\cdot m_{j}+\frac{1}{\gamma }\text{tan}\;\left(\frac{\pi }{2}\frac{W_{kj}^{\text{exc}}}{W^{\text{max}}}\right)\right)\right],$$ (61)

i.e., $\phi_{\vartheta }(W_{kj}^{\text{exc}})=m_{k}\cdot m_{j}+\frac{1}{\gamma }\text{tan}\left(\frac{\pi }{2}\frac{W_{kj}^{\text{exc}}}{W^{\text{max}}}\right)$. While this plasticity rule preserves the symmetry of recurrent weights and features the desired fixed points $W_{kj}^{\text{exc}}=W(c_{kj})$, it must be noted that there exists no theoretical guarantee for convergence under recurrent network dynamics.

### Interaction of time scales

The learning rates *η*_*b*_, *η*_*V*_ and *η*_*W*_ control the typical time scales for significant changes in the parameters ***b***, ***V*** and ***W*** ^exc^. In an online EM learning scenario, these changes are interrelated with the network’s spike response and variations in the external input. In total, four different processes are to be distinguished which jointly orchestrate the learning dynamics:

1. On the fastest time scale, the synaptic time constant *τ* sets the typical scale for inference and mixing during sampling from the posterior. For this study, we set *τ* = 10 ms.
2. On a slower time scale—let’s refer to it as *τ*_inp_ for this discussion—significant changes in the presented input statistics occur, i.e. the input vector ***y*** ∈ {0,1}^*N*^ switches to substantially different regions in the space of possible inputs during sampling from p*(***y***).
3. The variational E-step integrates the average network response 〈***z***〉_p*(***y***)*q*(***z*** ∣ ***y***)_, and thus, relies on a representative coverage of the input space. Therefore, changes in ***b***, which happen on a time scale $\tau_{\text{E}}\approx \eta_{b}^{-1}$, must be slower than the mixing of ***y*** ∼ p*(***y***).
4. Finally, the M-step adapts synaptic weights ***V*** and ***W*** ^exc^ based on a reliable E-step, and hence, $\tau_{\text{M}}\approx \eta_{V}^{-1}$ (or $\eta_{W}^{-1}$) need to be large compared to *τ*_E_.

In summary, from a strictly theoretical perspective we require *τ* ≪ *τ*_inp_ ≪ *τ*_E_ ≪ *τ*_M_. In practice, we typically find a factor 5–20 per ≪ -relation to be sufficient. For instance, the network responds on the time scale of few to tens of milliseconds; input statistics vary on the time scale of hundreds of milliseconds to seconds; intrinsic neuronal excitabilities adapt on the time scale of tens of seconds to minutes; and synapses change their weight on the time scale of minutes to hours.

### Illustration of learning with homeostatic intrinsic plasticity

In this subsection, we exemplify the contribution of the homeostatic intrinsic plasticity rule (13) to self-organized learning in a spiking network. Intuitively speaking, homeostatic plasticity compensates for an “unrightful” advantage that network neurons with strong input weights (corresponding to high intensity input patterns) gain over network neurons with weak input weights (corresponding to low intensity input patterns) by regulating the excitabilities *b*_*k*_ of the neurons. Mathematically speaking, the correct value of the *b*_*k*_’s is determined by the analytically calculated posterior (37): the strong-weight advantage in the term ***z*** ^⊺^ ***V y*** is counterbalanced by the normalization constants *A*_*k*_ in the term $z^{⊺}\cdot (\hat{b}-A)$. This leads to excitabilities $b_{k}={\hat{b}}_{k}-A_{k}$ of the neurons for exact inference according to Eq (42). While the *A*_*k*_’s can in principle be calculated from the synaptic weight values *V*_*ki*_, it remains unclear how a network neuron could obtain the required knowledge of all afferent weight values. This is where homeostatic intrinsic plasticity comes into play. It approximates the *A*_*k*_-contribution during the E-step and, thus, enables the network neurons to perform variational inference.

**Fig 6:**
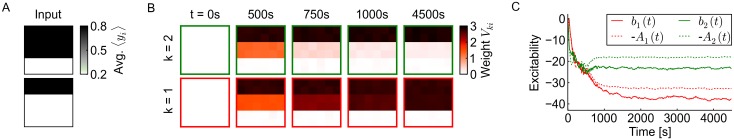
Illustration of learning with homeostatic intrinsic plasticity. (A) Input patterns that are particularly challenging for learning since the weak pattern (bottom) is fully contained in the strong pattern (top). (B) Evolution of afferent weights *V*_*ki*_(*t*) during learning with homeostatic intrinsic plasticity in a small two-neuron WTA network. (C) Evolution of excitabilities *b*_*k*_(*t*) alongside externally calculated normalizations *A*_*k*_(*t*). Homeostatic intrinsic plasticity enables the network neurons to track the contribution of the non-local values *A*_*k*_, and thereby implements a variational E-step.

We illustrate the contribution of Eq (13) to the learning process in a minimal example with only *K* = 2 network neurons and *N* = 6 × 6 input neurons, organized in a small WTA network. The presented input ***y***(*t*) ∼ p*(***y***) consists of only three prototypic activity patterns: the two patterns shown in Fig 6A with 〈*y*_*i*_〉 = 0.2 and 0.8 for “inactive” and “active” inputs respectively, and a uniform low-intensity background activity pattern with 〈*y*_*i*_〉 = 0.2. These somewhat artificial input patterns are an extreme and particularly challenging case for learning since the weak pattern (bottom) is fully contained in the strong pattern (top). As a consequence, a network neuron, which is specialized on the strong pattern (and hence maintains strong weights *V*_*ki*_) will always have a stronger input contribution ∑_*i*_ *V*_*ki*_ ⋅ *y*_*i*_ to the membrane potential *u*_*k*_ than a network neuron which is specialized on the weak pattern. The task of intrinsic plasticity is to continuously approximate the values *A*_*k*_ in the excitabilities *b*_*k*_ during learning in order to compensate the systematic (dis-)advantage.

In a computer simulation, all three input patterns were presented for an equal amount of time. Synaptic weights were initialized at *V*_*ki*_(*t* = 0) = 0, and homeostatic target activities were set to *m*_1_ = *m*_2_ = 0.32. The weight evolution *V*_*ki*_(*t*) for both network neurons is shown in Fig 6B. During an initial phase, lasting until *t* ≈ 500s, both neurons raised their input weights and integrated the average activity of both patterns. Then, WTA competition began to enforce a separation of the two patterns. At the end of learning, each network neuron had become an expert for one of the patterns (the low-intensity background pattern is covered by the default value *π*_0*i*_ = 0.2). In particular, the weak pattern had successfully been identified by the plastic network. The contribution of homeostatic intrinsic plasticity to this learning process can be seen in Fig 6C. Shown is the evolution of intrinsic excitabilities *b*_*k*_(*t*) for both neurons, alongside the analytically correct normalizations −*A*_*k*_(*t*) calculated offline from Eq (29). Homeostatic intrinsic plasticity approximately tracks the time-varying contribution of −*A*_*k*_(*t*)’s to the excitabilities $b_{k}={\hat{b}}_{k}-A_{k}+\beta_{k}$ during the variational E-step Eq (43).

The ability to track the normalizations *A*_*k*_(*t*) in the excitabilities *b*_*k*_(*t*) is essential for successful learning: In a repetition of the above experiment with intrinsic excitabilities fixed at *b*_*k*_ = −2, only one of the network neurons developed significant afferent weight values and entered a state of continuous bursting (〈*z*〉 = 0.980 for *t* > 4000s). The other neuron remained almost silent (〈*z*〉 = 0.002 for *t* > 4000s) with weights close to zero.

### Details to the computer simulations

All computer simulations were performed with custom Python [86] scripts, using a discrete time version for spiking neurons and synaptic plasticity with simulation time step *δt* = 1 ms and PSP time constant *τ* = 10 ms. Python scripts to reproduce the simulations are provided as supporting information (S1 Code). In order to avoid boundary effects at the edge of the network, a torus-like network topology is used, i.e., neurons at the left-hand edge are adjacent to neurons at the right-hand edge, and neurons at the top edge are adjacent to neurons at the bottom edge of the sheet. Inhibitory connections were non-plastic. Excitatory connections, if plastic, were restricted to positive weight values. The constraint to positive weights was imposed for the purpose of neuroscientific modeling, only. The theory for inference and learning supports positive and negative weights (incl. sign changes). In the following, we first describe the implementation of neurons and synapses as used in all simulations. Then we provide specific simulation details for each figure. An overview of used parameters is provided in Table 1.

**Table 1:** Parameters of the computer simulations.

Category	Parameter	Symb.	Fig 2	Fig 3	Fig 4	Fig 5	Fig 6	Comments
Simulation	Sim. time	*T*	2.5	10,000	25,000	10,000	5,000	Unit [s]
	Time step	*δt*	0.001	0.001	0.001	0.001	0.001	Unit [s]
Network	Network neurons	*K*	6	21	21	144	2	
	Geometry		6 × 1	7 × 3	7 × 3	12 × 12	2 × 1	
	Neuron distance		3	3 × 2	6 × 1	2 × 2	0	Relative to inp. space
	Rec. exc. conn. range		–	–	∞	∞	–	Max. norm; input space
	Rec. exc. conn. prob.		–	–	1	0.25	–	
	Inh. conn. dist.		3	3	1	4	1	Max. norm
	Inh. weight	*W* ^inh^	-100	-100	-100	-100	-100	
	Inputs	*N*	108	126	252	576	36	
	Geometry		18 × 6	21 × 6	42 × 6	24 × 24	6 × 6	
	Afferent conn.		6 × 6	6 × 6	6 × 6	6 × 6	6 × 6	
	PSP-/Ref.-time	*τ*	0.010	0.010	0.010	0.010	0.010	Unit [s]
	Backgr. act.	*π*_0*i*_	0.2	0.1	0.1	0.1	0.2	
Stimulus	Activity range		0.2/0.55	0.1/0.6	0.1/0.4	0.1/0.5	0.2/0.8	Min./Max. ⟨ *y*_*i*_ ⟩
	Pattern duration		–	200ms^1)^	250ms^2)^	100ms	1000s	During learning
Plasticity	Hom. target	*m*_*k*_	–	0.065	0.95/3	0.025	0.32	
	Init bias	*b*_*k*_	−1 − *A*_*k*_	-2	-1	-3	-2	at *t* = 0
	Init inp. weight	*V*_*ki*_	Eq (29)	0	0	0	0	at *t* = 0
	Init rec. weight	$W_{kj}^{\text{exc}}$	0 or 1	0	0	0	0	at *t* = 0
	Learn rate bias	*η*_*b*_	0	1	0.1	10	1.5	Unit [Hz]
	Learn rate inp. syns	*η*_*V*_	0	0.2	0.1	2	0.3	Unit [Hz]
	Learn rate rec. syns	*η*_*W*_	0	–	0.005^3)^	1	–	Unit [Hz]

^1)^Average duration of locally occurring patterns.

^2)^In case of an ambiguous cue; otherwise: 2×250ms.

^3)^0.05 for wake-sleep learning.

#### Stochastic neuron model (common to all figures)

We employed the simplest neuron model from [17] with an absolute refractory period. Neurons are characterized by their membrane potential *u*_*k*_ and a refractory time *τ* that matches the time constant the associated RV *z*_*k*_ is active after a spike. In discrete time, the active period lasts for $\tilde{\tau }≔\tau /\delta t=10$ time steps. The spiking probability in each time step reads $p(\text{spike})=\sigma (u_{k}-\text{log}\tilde{\tau })$ if the neuron is non-refractory. A neuron is non-refractory if *z*_*k*_ = 0 or if it is in its last active time step (to allow an uninterrupted active state, see [17]). Neurons in a network were updated sequentially such that state transitions of one cell are visible in the same time step to subsequently updated neurons. In the computer simulations, the update order was chosen randomly, in every time step. Just as for the continuous time neuron model of the Results section, networks that employ this discrete time neuron model are proven [17] to sample from the correct target distribution in the sense of Corollary 1. For a discrete time implementation of homeostatic intrinsic plasticity, we updated the excitability ***b*** in every time step according to *δb*_*k*_ = *δt* ⋅ *η*_*b*_ ⋅ (*m*_*k*_ − *z*_*k*_). This is a simple Euler integration of the continuous time plasticity rule.

In the limit *δt* → 0 and $\tilde{\tau }\to \infty$ while keeping *τ* = *const*., we obtain the continuous time neuron model from the discrete time model. In continuous time, the sequential update policy in the network disappears and all network neurons evolve in parallel. The Markov Chain that underlies continuous time implementations is expected to show even better mixing properties (in terms of biological real-time, not the number of time steps) than the discrete time model used in the simulations: In continuous time, if multiple local WTA neurons compete for explaining the input, an active neuron will (almost surely) switch back to the inactive state at the end of its active phase; the next spike of the local WTA population will then immediately be a correct sample from the local posterior distribution. In contrast, a discrete time neuron has a non-vanishing probability to re-spike in the last time step of its active phase, thereby generating strongly correlated samples. As a consequence, we expect that all results obtained from discrete time computer simulations remain valid without any restrictions in the continuous time limit.

#### Synaptic transmission and plasticity (common to all figures)

A spike of the i-th neuron elicits a rectangular post-synaptic potential (PSP) at the k-th neuron with duration *τ* and amplitude *V*_*ki*_ (*W*_*ki*_) for afferent (recurrent) connections. Synaptic transmission has zero-delay and is non-additive, and thus, PSPs encode the value of the pre-synaptic random variable times the synaptic weight at any time. In discrete time, PSPs last for $\tilde{\tau }$ time steps, accordingly. For a discrete time implementation of plasticity, we updated the weights ***V***, ***W*** ^exc^ in every time step according to *δV*_*ki*_ = *δt* ⋅ *η*_*V*_ ⋅ *z*_*k*_ ⋅ (*y*_*i*_ − *σ*(*V*_*ki*_ + *V*_0*i*_)) and $\delta W_{kj}^{\text{exc}}=\delta t\cdot \eta_{W}\cdot (z_{k}\cdot z_{j}-\phi )$. Here *ϕ* denotes the LTD term of wake-sleep learning and the approximate plasticity rule, respectively. Again, this is a simple Euler integration of the continuous-time plasticity rules.

#### Spiking input generation (common to all figures)

In all simulation, spiking input was presented to the network in form of Poisson spike trains with time varying firing rate. In accordance with the synaptic transmission in the network via non-additive, rectangular PSPs, an input RV *y*_*i*_(*t*) has value 1 if a spike had occurred in the i-th input channel within (*t* − *τ*, *t*], and value 0 otherwise. Firing rates are chosen such that the expected value of *y*_*i*_ matches a target activity *x*_*i*_, i.e., 〈*y*_*i*_〉 = *x*_*i*_. The specific target values *x*_*i*_ used for the simulations are provided below for each figure. In order to achieve 〈*y*_*i*_〉 = *x*_*i*_ in a discrete time simulation, the spiking probability of an input is set to $p_{i}≔p(\text{spike in}\;\delta t)=1-{\left(1-x_{i}\right)}^{1/\tilde{\tau }}$ for each time step. This assignment originates from the following thought. An input is inactive when there was no spike in the last $\tilde{\tau }$ time steps, i.e., $p(y_{i}=0)={\left(1-p_{i}\right)}^{\tilde{\tau }}$. The expected value 〈*y*_*i*_〉 is thus given by $\langle \;y_{i}\;\rangle =p(y_{i}=1)=1-p(y_{i}=0)\;\overset{!}{=}\;x_{i}$. Solving for *p*_*i*_ yields the above assignment. In the limit *δt* → 0 such that $\tau =\tilde{\tau }\cdot \delta t=const.$, these spiking dynamics yield a Poisson process with firing rate log[(1 − *x*_*i*_)^−1/*τ*^].

#### Figure 2: Sheets of spiking neurons can perform Bayesian inference on distributed spiking input

The network consists of *N* = 18 × 6 input neurons and *K* = 6 network neurons. Each network neuron receives local input from 6 × 6 inputs, with local connections being shifted by 3 between neighboring network neurons. Neurons with overlapping input inhibit each other, resulting in nearest-neighbor inhibition. In addition, three neuron pairs ((*k*, *j*) = (1, 3), (3, 5) and (4,6)) maintain excitatory recurrent connections of weight $W_{kj}^{\text{exc}}=W_{jk}^{\text{exc}}=1$. Preferred local activity patterns *x*_*kj*_ ∈ (0.2,0.55), 1 ≤ *k* ≤ 6 and 1 ≤ *j* ≤ 36, were drawn for each network neuron from a uniform distribution. Background activity was set to *π*_0*i*_ = 0.2, and afferent synaptic weights were set to match the patterns according to Eq (29). Neuronal excitabilities were set to $b_{k}={\hat{b}}_{k}-A_{k}$ with ${\hat{b}}_{k}=-1$ and *A*_*k*_ = ∑_*i*_ *A*_*ki*_ being calculated for each neuron according to Eq (29).

**Fig 7:**
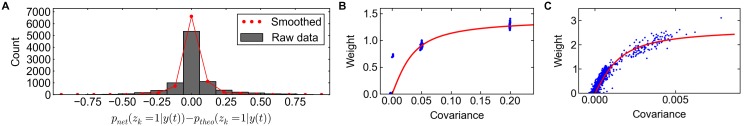
Sampling quality and heuristic learning rule. (A) Sampling quality of the spiking network. Red: Histogram over the difference between the traces in Fig 2D for every neuron and time point. For visual clarity, the data in Fig 2D had been smoothed with a 20ms box kernel. Gray: Histogram over the non-smoothed, raw data. (B) Recurrent plasticity function in the setup of Fig 4. Weights obtained with wake-sleep learning vs. the covariance of network variables (blue dots), alongside the fitted plasticity function *W*(*c*_*kj*_)(red line). (C) Same for Fig 5.

Spiking input was generated with the aim that the input distribution p*(***y***) closely resembles the model distribution *p*(***y*** ∣ ***θ***) of the network: At each location and time point, at most one local input pattern *x*_*kj*_ is active. If pattern *x*_*kj*_ is active, it governs the firing rate of all those inputs that are connected to neuron *k*, i.e., 〈*y*_*i*_〉 = *x*_*kj*_ for *i* = [(18 ⋅ (*k* − 1) + (*j*−1) − 6) mod *N*] + 1. The presence of an input pattern is indicated by colored spikes in Fig 2E. If no dedicated pattern is active, inputs fire with the background activity, i.e., 〈*y*_*i*_〉 = *π*_0*i*_ (gray spikes). The data shown in Fig 2 and evaluated Fig 7A covers 1.5s simulation time. The total simulation time was 2.5s with a short period before and after the shown data being discarded. This serves to provide a burn-in phase for the sampling network, and to prevent boundary artifacts when smoothing the posterior marginals. In panel D, the posterior marginals *p*_net_ were estimated from the network response ***z***(*t*) during 1000 simulation runs, all with exactly the same input (spike-level identity). The correct posterior *p*_theo_ is given by Eq (37). For visual clarity, the traces of the posterior marginals have been smoothed with a 20ms box kernel.

A comparison of non-smoothed data is provided in Fig 7A. The figure shows a more systematic analysis of the sampling quality of the spiking network by means of a histogram over the mismatch (*p*_*net*_(*z*_*k*_ = 1 ∣ ***y***(*t*)) − *p*_*theo*_(*z*_*k*_ = 1 ∣ ***y***(*t*)). The red dotted counts were evaluated on the smoothed data of Fig 2D; the gray bars depict the non-smoothed (raw) data, evaluated on a millisecond basis. This quantitative analysis confirms the excellent approximation quality of the sampling network. In conclusion, the data shown in Figs 2D and 7A indicate that the sampling network can calculate and represent the general structure as well as quantitative specificities of the time-varying posterior distribution with high accuracy. However, small differences between the traces in Fig 2D are visible. Most notably, rapid and sharp peaks in the posterior, which arise from pronounced but transient jumps in the input, are not fully integrated by the network. The origin of these deviations is of stochastic and systematic nature. Stochastic fluctuations arise from the general sample-based representation. For time-varying input signals ***y***(*t*), only few independent samples can be drawn from the target posterior distribution *p* (***z*** ∣ ***y***(*t*), ***θ***) under (almost) stable conditions, i.e., before the target distribution changes. Any representation based on a limited number of samples can approximate the posterior only with limited precision. Systematic deviations result from incomplete convergence of the Markov chain. The Markov chain, that underlies the network dynamics, is guaranteed to converge to the correct equilibrium distribution *p* (***z*** ∣ ***y***, ***θ***) only for any constant input ***y*** in the limit *t* → ∞. For time-varying input ***y***(*t*), convergence of the network will typically “lag behind” the “moving target” *p* (***z*** ∣ ***y***(*t*), ***θ***). Theoretical work [18] has shown that the network distribution converges exponentially fast to its equilibrium in almost arbitrary network architectures. Notably, the local WTA architecture is expected to facilitate mixing of the Markov chain since typically only a few competing neurons will attempt to fire in response to the presented input at the same time.

#### Figure 3: Emergence of probabilistic local experts through synaptic plasticity

The network consists of *N* = 21 × 6 input neurons and *K* = 7 × 3 network neurons. Network neurons are organized in 7 local populations. The 3 neurons within a population share the same 6 × 6 field of afferent connections. Afferent fields of neighboring populations are shifted by 3 (measured in the domain of input neurons). Due to the torus-like topology, every network neuron has overlapping inputs with 8 other network neurons (2 in its population and 2 × 3 in the neighboring populations). This is the range of lateral inhibition.

Spatio-temporal spiking input (that determines the samples ***y*** and thus p*(***y***)) was generated as follows. For each of the 7 afferent field locations, 3 random activity patterns $x_{lj}^{p}$, (1 ≤ *p* ≤ 3, 1 ≤ *l* ≤ 7, 1 ≤ *j* ≤ 36), were drawn. To facilitate the generation of locally different activity patterns, each input location was drawn from a Dirichlet distribution and scaled to the activity range [0.1,0.6]: $(x_{lj}^{1},x_{lj}^{2},x_{lj}^{3})∼0.5\cdot \text{Dir}(0.3,0.3,0.3)+0.1$. Whenever a local activity pattern $x_{l}^{p}$ was presented, Poisson spike trains were generated such that $\langle \;y_{i}\;\rangle =x_{lj}^{p}$ with *i* = (18 ⋅ *l* + *j* mod *N*) + 1. The presence of local activity patterns $x_{l}^{p}$ was determined as follows. Three chains *c* = 1,2,3 were started at time *t* = 0. Each chain can either be active or inactive. If it is active, it appears at a location *l* and presents one of the local activity patterns $x_{l}^{p}$. Initially, all chains were inactive and the initial duration of inactivity (in ms) was drawn for each chain from a Gamma distribution Γ(*k* = 10, *θ* = 10), leading to an average initial inactivity of 100 ms. Whenever inactivity of a chain ends, it turns active for a duration (in ms) drawn from Γ(*k* = 10, *θ* = 20), leading on average to 200 ms duration of activity. When a chain turns active, a random pattern *p* is drawn uniformly, and a random location *l* is drawn such that the invoked activity pattern $x_{l}^{p}$ does not overlap with the local activity pattern of a different currently active chain. Such a valid location always exists in the given architecture. After a chain’s active phase, it turns inactive again for a duration (in ms) drawn from Γ(*k* = 10, *θ* = 10). Inputs, that are not covered by a currently active chain, maintain a background activity with 〈*y*_*i*_〉 = *π*_0*i*_ = 0.1. This process leads to spatially non-overlapping, but temporally interleaved input spike patterns as shown in Fig 3F.

The network was exposed to this spiking input for 10000 s. Afferent weights *V*_*ki*_ and intrinsic excitabilities *b*_*k*_ were plastic; recurrent connections $W_{kj}^{\text{exc}}$ and $W_{kj}^{\text{inh}}$ were non-plastic, and $W_{kj}^{\text{exc}}=0$.

Details to the plotting: Panel B shows the activity patterns $x_{lj}^{p}$, *p* = 1,2,3, for the input region highlighted in panel A. For estimating the log-likelihood in panel C, *S* = 10000 input samples ***y*** ^*s*^ were randomly drawn from the training data as a proxy for p*(***y***). Furthermore, the network has 337 possible ***z***-states that respect the inhibition structure. Thus, the log-likelihood can be calculated via $ℒ(\theta )\approx \frac{1}{S}\sum_{s}\;\text{log}\sum_{z}p(z)\cdot \prod_{i}p_{}\left(y_{i}^{s}\;|\;z,\;\theta \right)$, with $p_{}\left(y_{i}^{s}\;|\;z,\;\theta \right)$ given by Eq (25), for any afferent weight configuration ***V*** that emerges over the course of learning. The joint distribution depends on the parameters ***W*** ^inh^, ***V*** and $\hat{b}$. While the synaptic parameters ***W*** ^inh^, ***V*** are directly accessible in the spiking network, the biases $\hat{b}$ in the prior must be determined differently. We calculated the biases $\hat{b}$ offline such that the prior exhibited the homeostatic target activity, i.e. 〈*z*_*k*_〉_*p*(***z*** ∣ ***θ***)_ = *m*_*k*_ for all *k*. This is a canonical choice since these are the biases $\hat{b}$ a Bayesian observer would determine from observing the network response. In panel D, optimal weights were calculated from the generating input patterns $x_{l_{j}}^{p}$ according to Eq (29). This is possible since the data distribution p*(***y***) is structurally similar to the model distribution *p*(***y*** ∣ ***θ***). For each of the seven input locations, each of the three local network neurons was assigned to the best matching pattern of optimal weights. The assignment was unambiguous, since each network neuron had clearly specialized on one of the local input patterns, and determines the one-to-one mapping between learned weights *V*_*ki*_ and optimal weights plotted in panel D. For panels E and G, four additional simulations were run with network parameters (***V***(*t*), ***b***(*t*)) taken from different training time points *t* = 0 s, 1000 s, 3000 s, 10000 s. Identical 100s-spike patterns were presented to the network in these four simulations. Panel G shows [2.5]*s* of the input spike pattern alongside the network response for these simulations. Panel F shows the corresponding afferent weights ***V***(*t*) for the three highlighted network neurons. For the 2-dimensional linear projection in panel E, the input states ***y***(*t*) of each 100s-simulation were sampled every 10 ms (i.e. 10000 data points per scatter plot) and projected onto the 2d plane. The color of each data point is determined by the network response: red, green, blue if one the neurons marked in panel A responded; and gray otherwise. The projection plane is spanned by the two leading principle components (PCA) of those input samples the three highlighted network neurons responded to at the end of learning, i.e., the PCA is based on the colored samples in the rightmost panel. This biased selection only concerns the choice of the projection plane with the aim to visually discern the clusters of interest; the plotted data points are unbiased.

#### Figure 4: Plastic recurrent synapses integrate structural knowledge

The network consists of *N* = 7 × 6 × 6 input neurons and *K* = 7 × 3 network neurons. Network neurons are organized in 7 local populations. The 3 neurons within a population share the same 6 × 6 field of afferent connections. The afferent fields of different populations are disjoint. Network neurons within the same population share lateral inhibition. Network neurons, which belong to different populations, maintain excitatory recurrent connections $W_{kj}^{\text{exc}}\geq 0$.

For the spiking input, a simpler temporal input structure than in Fig 3 was used since the focus of this simulation was set at the correlation structure of the input beyond the range of individual input fields. There are three local activity patterns $x_{l}^{p}$, *p* = 1,2,3, referred to as the red, green and blue pattern, each consisting of “vertical stripes” at shifted locations: $x_{lj}^{p}=0.4$ if ⌊(*j*−1)/⌋ *mod* 3 = (*p*−1), and 0.1 otherwise, with ⌊⋅⌋ denoting the floor function. To generate a global activity pattern *x*, two (potentially identical) cue patterns $x_{l}^{p}$ were picked for the outermost locations *l* = 1 and *l* = 7. Then the inner locations *l* = 2—6 were filled with a consistent valid pattern, with “validity” referring to the condition to show a pattern that differs from the cues. Thus, in case of two different cue patterns, the choice of the inner pattern was fully determined (e.g. a red and green cue leads to blue inner patterns); in case of two identical cues, the inner pattern could show either of two valid patterns with all inner locations showing the same pattern (e.g. a double-green cue leads to either all-red or all-blue inner patterns). Hence, both cues (*l* = 1 and *l* = 7) must be taken into consideration to determine the validity of inner patterns (2 ≤ *l* ≤ 6) during inference, and all inner patterns are supposed to be of equal type. As in previous simulations, input Poisson spike trains were generated such that the average value 〈***y***〉 matched the activity pattern ***x***. Input activity patterns ***x*** were presented for a fixed duration before the global activity pattern switched: The patterns iterated over the nine possible cue combinations. In case of differently colored cues the global pattern was presented for 500 ms; in case of identical cues the two valid global patterns were presented for 250 ms each. Thus all cue combinations were presented for an equal amount of time. After all cues were presented, the presentation was repeated.

For the learning experiments, the network was exposed to this spiking input for 25000 s, i.e., each cue combination was presented 25, 000 s/0.5 s/9 ≈ 5, 500 times. In total, three learning experiments were conducted with recurrent plasticity being (a) governed by the theoretically optimal wake-sleep rule, (b) governed by the simple heuristic rule, (c) switched off. In all simulations, afferent weights *V*_*ki*_ and intrinsic excitabilities *b*_*k*_ were plastic. In (a) and (b) recurrent connections $W_{kj}^{\text{exc}}$ were plastic and restricted to positive (excitatory) weight values. For (a) “wake-sleep learning”, the theoretically derived learning rule (53) was used with *η*_*W*_ = 0.05, and prior samples being drawn from an independent sampling network which shared its recurrent weights $W_{kj}^{\text{exc}}$ with the learning network but maintained independent (homeostatically regulated) biases and was not exposed to any input. After learning, the covariances *c*_*kj*_ were calculated from the posterior samples ***z***(*t*) of the last 1000s of the simulation. To obtain data points ($W_{kj}^{\text{exc}},c_{kj}$) for fitting the function *W*(*c*_*kj*_), only excitatory synapses were considered that had a weight $W_{kj}^{\text{exc}}>0.01$ at the end of learning. This is to prevent distortions in the fit due to synapses between negatively correlated neurons that would have developed negative weights during wake-sleep learning (but were bounded to $W_{kj}^{\text{exc}}\geq 0$ in the simulation). Fitting the function (60) to this data yielded *W*_max_ = 1.4113 and *γ* = 31.606 for the free parameters. Data points and fitted function are shown in Fig 7. For (b) “heuristic learning”, the fitted learning rule was used with *η*_*W*_ = 0.005. All recurrent weights $W_{kj}^{\text{exc}}$ converged to stable values. Weights connecting inner neurons (2 ≤ *l* ≤ 6), that had specialized on equal activity patterns, settled at $W_{kj}^{\text{exc}}\approx 1.27$. Weights between cue neurons and compatible inner neurons settled at $W_{kj}^{\text{exc}}\approx 0.90$. Weights between cue neurons responsive to the same pattern settled at $W_{kj}^{\text{exc}}\approx 0.32$. All other weights settled close to zero ($W_{kj}^{\text{exc}}<0.003$).

For the demonstration of inference in face of incomplete observations (panels E and F), activity patterns ***x*** were generated as follows. Two cues were chosen at the outer locations. These cues had increased contrast to ensure that the spike pattern at the cue locations was unambiguous: $x_{lj}^{p}=0.6$ if ⌊ (*j*−1)/3⌋ mod 3 = (*p*−1), and 0.1 otherwise (for *l* = 1 and *l* = 7). All inner locations *l* = 2—6 showed uninformative uniform activity of moderate intensity:*x*_*lj*_ = (0.6 + 0.1)/2 = 0.35 for all *j*. We refer to the uninformative patterns as “gray” patterns. For each cue combination, Poisson spike trains with 〈***y***〉 = ***x*** were presented to the network for 100 s. The vertical bars in panel E and F show the mean activity 〈*z*_*k*_〉 of each network neuron during the simulation given an unambiguous cue (panel E) and an ambiguous cue (panel F).

The log-likelihood ℒ(***θ***(*t*)) in panel D was estimated as follows.*S* = 10000 input samples ***y*** ^*s*^ were randomly drawn from the training data as a proxy for p*(***y***). Furthermore, the network has 4^7^ = 16384 possible ***z***-states (zero or one active neuron in each local population). Thus, the log-likelihood can be calculated via $ℒ(\theta )\approx \frac{1}{S}\sum_{s}\;\text{log}\;\sum_{z}p(z)\cdot \prod_{i}p_{}\left(y_{i}^{s}\;|\;z,\;\theta \right)$ for each time point *t* and each network type (a)-(c). The joint distribution depends on the parameters ***W*** ^exc^, ***W*** ^inh^, ***V*** and $\hat{b}$. While the synaptic parameters ***W*** ^exc^, ***W*** ^inh^, ***V*** are directly accessible in the spiking network, the biases $\hat{b}$ in the prior must be determined differently. We calculated the biases $\hat{b}$ offline for each weight configuration (***W*** ^exc^(*t*), ***W*** ^inh^) such that the prior matched the homeostatic target activity, i.e. 〈*z*_*k*_〉_*p*(***z*** ∣ ***θ***)_ = *m*_*k*_. The log-likelihood is shown only for the first 10,000s of the simulation in order to highlight the early stage of learning.

#### Figure 5: Emergence of excitatory subnetworks in neural sheets

The network consists of *N* = 24 × 24 input neurons and *K* = 12 × 12 network neurons. Network neurons are organized in a sparse grid with twice the distance of inputs. Each network neuron maintains afferent connections with 6 × 6 inputs, such that the input field of neighboring neurons is shifted by two. Lateral inhibition has a range of 2 (in the network grid; maximum norm). Additionally, any pair of network neurons (beyond the range of inhibition) maintains a plastic reciprocal excitatory connection $W_{kj}^{\text{exc}}$ with 25% probability. All existing excitatory connections are plastic with initial values *V*_*kj*_(*t* = 0) = 0 and $W_{kj}^{\text{exc}}(t=0)=0$. “Non-existing” recurrent excitatory connections have a weight fixed at $W_{kj}^{\text{exc}}=0$. Note that non-existing connections are fully supported by the theory for inference and learning: reducing the number of plastic synapses in the network means to reduce the number of free parameters ***θ*** of the generative model *p*(***y***, ***z*** ∣ ***θ***). The derivative (53) in the direction of existing weights $W_{kj}^{\text{exc}}$ remains unaffected. Thus, the reduction only decreases the expressive power of the generative model.

Spiking input is composed of three prototypic rate patterns $x_{i}^{p}$, 1 ≤ *p* ≤ 3 (grid, diagonal stripes, checkerboard), occurring locally at random locations (cp. panel B). Activity patterns are binary with a high rate of $x_{i}^{p}=0.5$ and a low rate of $x_{i}^{p}=\pi_{0i}=0.1$. The diameter of local patterns is variable, but exceeds the 6 × 6 input field of individual network neurons. Two randomly selected (and possibly equal) patterns are presented simultaneously at non-overlapping locations. New patterns and locations are drawn every 100ms during training. All inputs not covered by a pattern fire with the background activity 〈*y*_*i*_〉 = 0.1. Homeostatic target activations *m*_*k*_ = 0.025 are chosen such that the network explains on average approx. 130 inputs; this is roughly half of the average area covered by rate patterns $x_{i}^{p}$, i.e., ca. 50% of the input is on average being explained by the network.

The network was first trained with wake-sleep learning and variable learning rates until all parameters had converged to stable values. The simple learning rule was fitted to the resulting ($W_{kj}^{\text{exc}},c_{kj}$)-pairs just as for Fig 4, yielding parameters *W*_max_ = 2.7049 and *γ* = 733.69. See Fig 7 for data and fitted function *W*(*c*_*kj*_). The high value of the sensitivity *γ*, compared to Fig 4, likely originates from the generally much lower covariance *c*_*kj*_, which in turn arises from the lower average network activity *m*_*k*_.

For the subsequent spiking network simulation with the simple plasticity rule, a new network with different connected pairs of network neurons was generated. Thus, the extracted plasticity rule is only tailored to the general learning setup, but not to a specific network instance. The total simulation time was *T* = 10,000s, i.e, the network was presented with 100,000 input examples. Learning rates were set to quite high values (*η*_*b*_ = 10, *η*_*V*_ = 2, *η*_*W*_ = 1) since the focus of this simulation was on the general structure of emerging weight configurations, rather than on numerical precision. After training, recurrent weights covered the entire range of positive weight values with $\text{max}[W_{kj}^{\text{exc}}]=2.275$. To verify that the emergent weight configuration was stable even in face of high learning rates, the simulation was continued for another 10,000s, showing no signs of instability.

Details to the plotting: For panels C and D, the trained network was exposed to spiking input with patterns switching every 500ms. This was to obtain more stable estimates for the “expected input” 〈***y*** ^gen^〉 in panel D. For panel E, all neurons could unambiguously be labeled to be responsive to one of the three local patterns, since their afferent weights *V*_*ki*_ showed an evident preference for either one of them. Panel F uses the euclidean distance in the lattice coordinates of the network neurons, i.e., directly neighboring network neurons have distance one.

## Supporting Information

S1 Code (ZIP)

S1 Video (AVI)
