# Supplementary figures and images for: Distributed Bayesian Computation and Self-Organized Learning in Sheets of Spiking Neurons with Local Lateral Inhibition
Source: PLoS One. 2015 Aug 18;10(8):e0134356. doi: 10.1371/journal.pone.0134356 (PMC4540468; doi:10.1371/journal.pone.0134356)

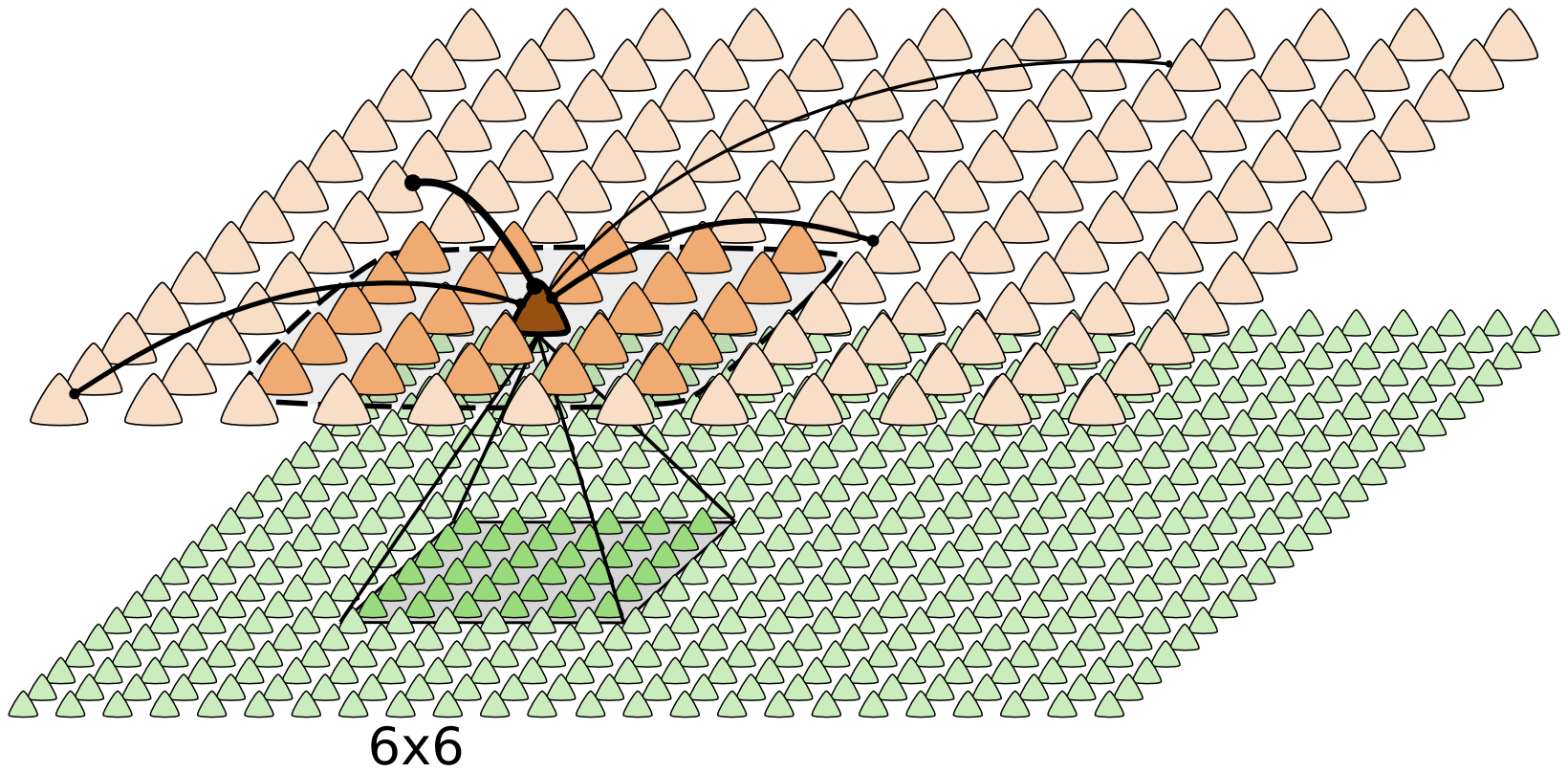

Supplement: S1 Code — (ZIP) [file pone.0134356.s001.zip › Code_Bill_et_al_2015/Fig5/fig/sketch.png]

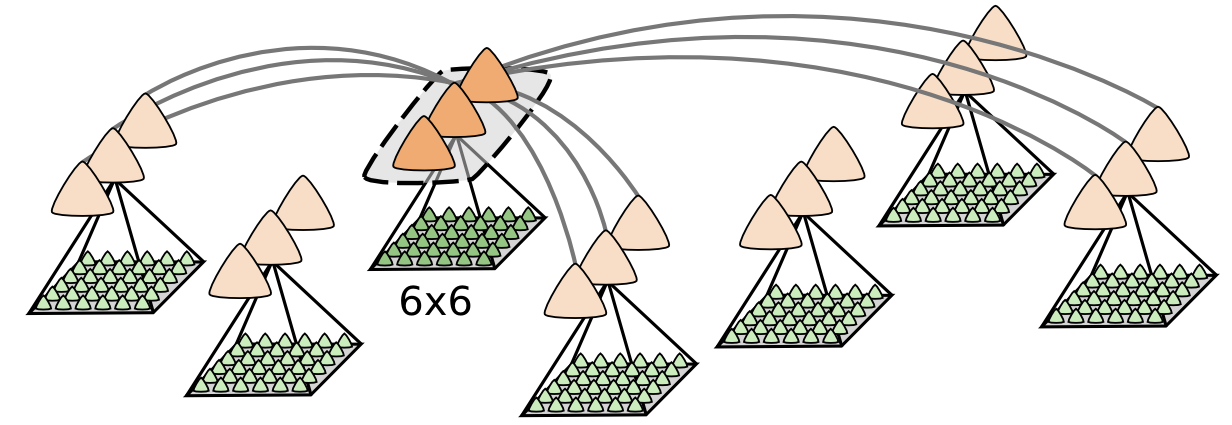

Supplement: S1 Code — (ZIP) [file pone.0134356.s001.zip › Code_Bill_et_al_2015/Fig4/fig/subplot_A.png]

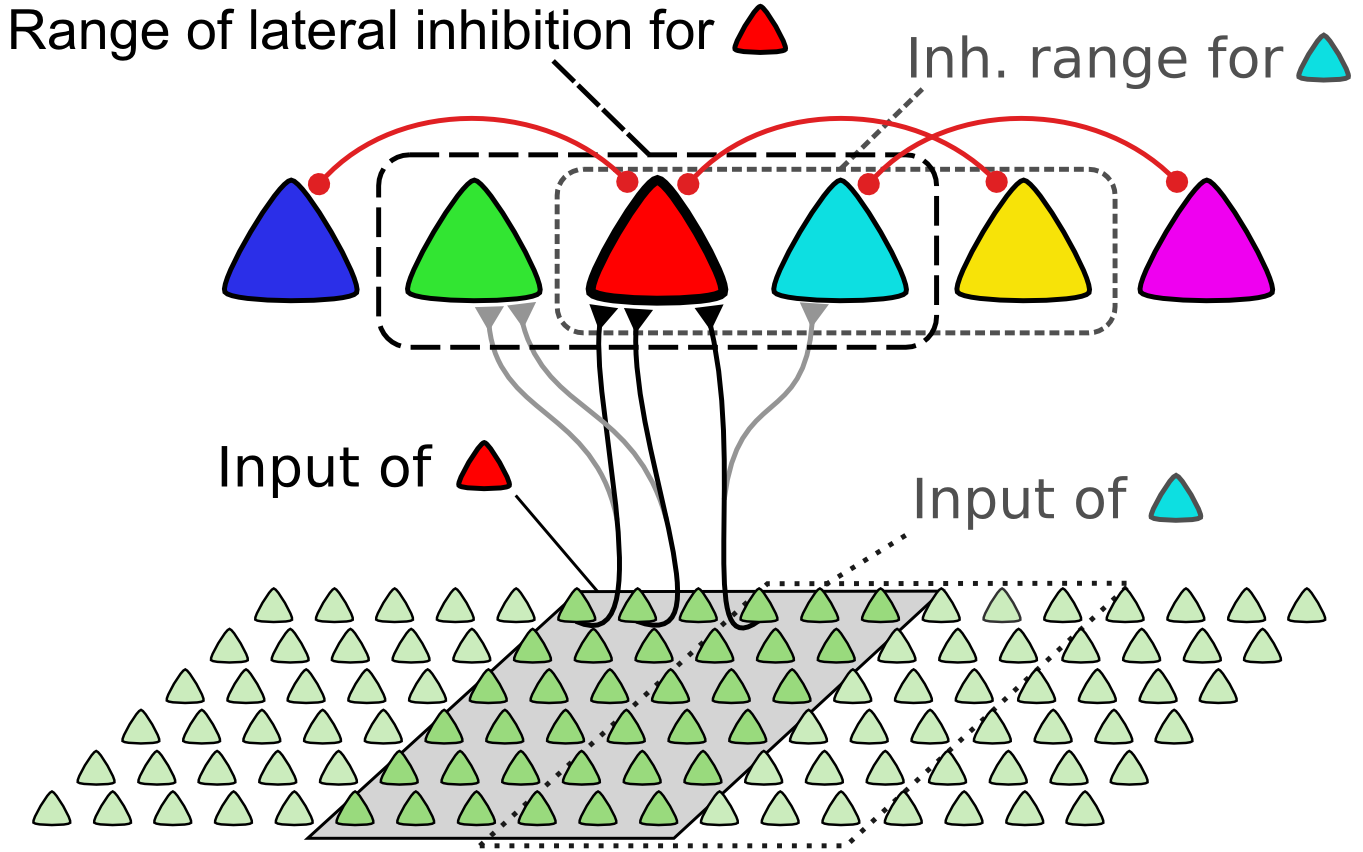

Supplement: S1 Code — (ZIP) [file pone.0134356.s001.zip › Code_Bill_et_al_2015/Fig2/fig/fig2c.png]

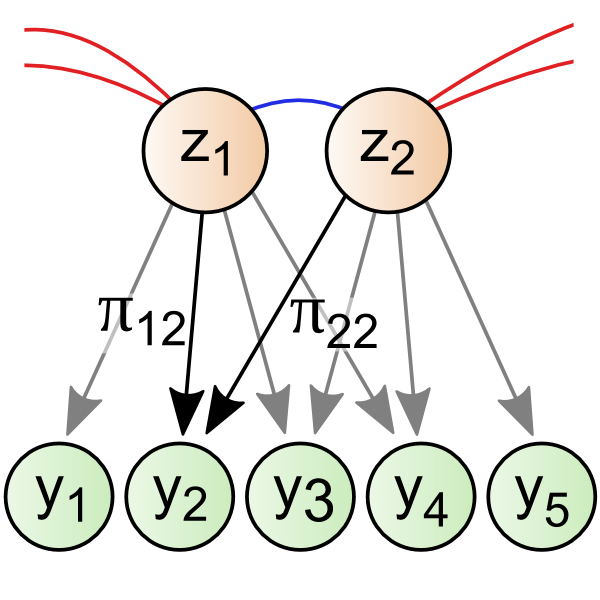

Supplement: S1 Code — (ZIP) [file pone.0134356.s001.zip › Code_Bill_et_al_2015/Fig2/fig/fig2a.png]

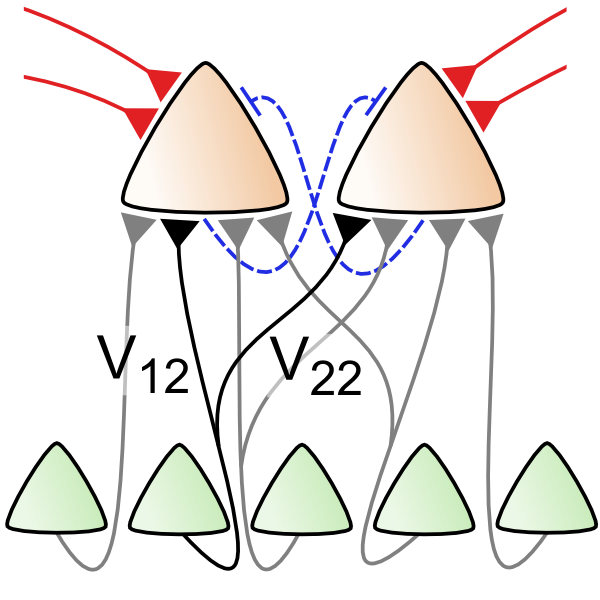

Supplement: S1 Code — (ZIP) [file pone.0134356.s001.zip › Code_Bill_et_al_2015/Fig2/fig/fig2b.png]

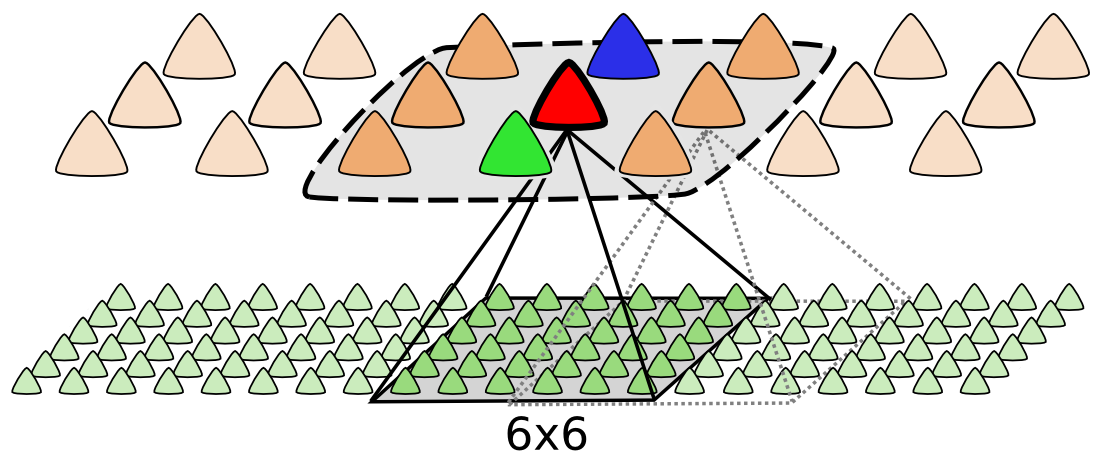

Supplement: S1 Code — (ZIP) [file pone.0134356.s001.zip › Code_Bill_et_al_2015/Fig3/fig/fig3a.png]
